# Anticancer drug sensitivity prediction in cell lines from baseline gene expression through recursive feature selection

**DOI:** 10.1186/s12885-015-1492-6

**Published:** 2015-06-30

**Authors:** Zuoli Dong, Naiqian Zhang, Chun Li, Haiyun Wang, Yun Fang, Jun Wang, Xiaoqi Zheng

**Affiliations:** 1 Department of Mathematics, Shanghai Normal University, Shanghai, China; 2 Department of Mathematics, Bohai University, Jinzhou, China; 3 Department of Bioinformatics, School of Life Science and Technology, Tongji University, Shanghai, China

**Keywords:** Drug sensitivity prediction, Feature selection, Recursive feature elimination

## Abstract

**Background:**

An enduring challenge in personalized medicine is to select right drug for individual patients. Testing drugs on patients in large clinical trials is one way to assess their efficacy and toxicity, but it is impractical to test hundreds of drugs currently under development. Therefore the preclinical prediction model is highly expected as it enables prediction of drug response to hundreds of cell lines in parallel.

**Methods:**

Recently, two large-scale pharmacogenomic studies screened multiple anticancer drugs on over 1000 cell lines in an effort to elucidate the response mechanism of anticancer drugs. To this aim, we here used gene expression features and drug sensitivity data in Cancer Cell Line Encyclopedia (CCLE) to build a predictor based on Support Vector Machine (SVM) and a recursive feature selection tool. Robustness of our model was validated by cross-validation and an independent dataset, the Cancer Genome Project (CGP).

**Results:**

Our model achieved good cross validation performance for most drugs in the Cancer Cell Line Encyclopedia (≥80 % accuracy for 10 drugs, ≥ 75 % accuracy for 19 drugs). Independent tests on eleven common drugs between CCLE and CGP achieved satisfactory performance for three of them, i.e., AZD6244, Erlotinib and PD-0325901, using expression levels of only twelve, six and seven genes, respectively.

**Conclusions:**

These results suggest that drug response could be effectively predicted from genomic features. Our model could be applied to predict drug response for some certain drugs and potentially play a complementary role in personalized medicine.

**Electronic supplementary material:**

The online version of this article (doi:10.1186/s12885-015-1492-6) contains supplementary material, which is available to authorized users.

## Background

Though having quite similar clinical symptoms, different patients may have different responses to the same drug or therapy. So personalized medicine, which makes medical decisions based on patients’ genetic content, becomes the main direction of the future medical science. In order to develop and access targeted therapies for individuals, one must resort to the lengthy and expensive process of drug development and validation in clinical trials, the most direct way to assess drug efficacy and toxicity. But the scarcity of resources limited this scheme in practical applications. One possible solution to this problem is to directly measure the sensitivity of a patient’s tumor cells to a drug of interest in two/three-dimensional in-vitro cultures [1] or in-vivo models such as mouse xenograft and genetically engineered mouse models [2]. This approach has the potential of capturing most of the relevant biological features of a patient’s tumor, and therefore providing better models to test drug sensitivity. However, such an approach is costly, time consuming and hardly scalable to screen dozens or hundreds of drugs in parallel.

With the development of the high-throughput technology in the past few decades, an alternative scheme was proposed by several research groups to build genomic predictors of drug response from large panels of cancer cell lines [3–8]. Most of these methods are based on gene expression profile. For instance, Staunton et al. developed a weighted voting classification model to predict anticancer drug sensitivity based on gene expression profile of NCI-60 data [9]. Based on the same dataset, Riddick et al. built an ensemble regression model using Random Forest [10]; Lee et al. developed a co-expression extrapolation algorithm by comparing the differences of gene expression between sensitive and resistant cell lines [11]. Meanwhile, other researchers focused on a specific type of cancer owing to the diversity of different cancer types (Biomarkers of a certain drugs for different cancers are different). For example, Holleman et al. studied gene expression patterns in drug-resistant leukemia cells, which showed that the combination of resistant gene expression is closely related to the risk of recurrence of disease [12]. In addition to gene expression, some researchers explored the relationships between chemotherapy sensitivity and epigenetic modifications. For example, Shen et al. used nucleotide sequences of methylation to predict drug response in cancer cells via a series of methylation markers. Although many biomarkers have been detected, these methods are still limited by the relatively small sample size. In order to further clarify the relationship between anticancer drug sensitivity and genomic instability, researchers recently collected a large genetic data set of more than 1000 human tumor cell lines and their pharmacological responses of 24 and 138 anticancer drugs [3, 4]. They both applied an elastic net model to predict anticancer drug sensitivity based on genomic instability data including gene mutation, variation of DNA transcription, and cancer-related gene translocation.

However, from the practical perspective, patients may care more about whether a drug will work for them or not (sensitive or insensitive), rather than a specific value. In such case, anticancer drug sensitivity prediction becomes a binary classification problem instead of a regression problem, where genetic annotations are served as features and response indicator is the classification category. If some gene signatures are detected to be responsible for drug sensitivity, then one can resort some machine-learning tools to characterize these signatures of a patient based on high throughput profiling and predict its sensitivity to a given drug. Towards this aim, we first classified all cell lines in CCLE into three groups according to their normalized drug response values (activity area). After recursive feature selection and parameter optimization through cross validation, an SVM model was built for each drug in the CCLE dataset. 10-fold cross validation indicated that 10 of 22 drugs performed satisfactory performance with model accuracy (the predictive performance of the SVM model) more than 80 %. An independent test on CGP showed that 3 of 11 common drugs between CCLE and CGP achieved a good result in terms of IC_50_. This result reconfirmed the inconsistency of therapeutic response for some drugs between these two data sets [13]. The generation of genomic predictor of drug response in the preclinical setting as the model proposed in our study could potentially accelerate the emergence of “personalized” therapeutic regimens [14] and therefore improve cancer therapy.

## Methods

### Ethics statement

We declare that this study does not involve any ethical issues and the research is independent and impartial.

### Anticancer drug sensitivity

In order to develop robust genomic predictor of response to anticancer drugs, we collected, curated, and annotated published data sets of two recent large-scale preclinical studies, namely cancer cell line encyclopedia (CCLE) [3] and the cancer genome project (CGP) [4].

#### CCLE

Consists of a large scale of genomic data, i.e., gene expression, mutation status and copy number alteration for 947 human cancer cell lines, as well as 8-point dose–response curves for 24 chemical compounds across 479 cell lines. We used the area under dose–response curves (termed as activity area in [3]) to evaluate the sensitivity of drug to a given cell line. Compared to the IC_50_ and EC_50_, activity area could capture the efficacy and potency of a drug simultaneously. All cell lines in this dataset were cultured in RPMI or DMEM with 10 % fetal bovine serum [15, 16].

#### CGP

The Cancer Genome Project used the human genome sequencing and high-throughput mutation detection techniques to identify somatically acquired sequence variants/mutations and hence identify genes critical to the development of human cancers (a compilation of gene expression, chromosomal copy number, and massively parallel sequencing data from 947 human cancer cell lines). Cell line drug sensitivity was measured as the concentration at which the drug inhibited 50 % of the cellular growth (IC_50_) [4]. All cell lines were grown in RPMI or DMEM/F12 medium supplemented with 5 % FBS and penicillin/streptavidin, and maintained at 37 °C in a humidified atmosphere at 5 % CO2.

Drug response data used in this paper were publicly available from the CCLE (www.broadinstitute.org/ccle/) and CGP (www.cancerrxgene.org/) websites. Raw gene expression profiles (Affymetrix CEL format) for CCLE and CGP cell lines were freely retrieved from the CCLE website and ArrayExpress under the accession number E-MTAB-783, respectively.

### Sample classification based on drug sensitivity

Drug sensitivity values (activity area in CCLE) were first normalized to zero mean and unit variance across all treated cell lines. For each drug, cell lines with normalized activity area at least 0.8 standard deviations (SDs) above the mean were defined as sensitive to the compound, whereas those with activity area at least 0.8 SDs below the mean were defined as resistant. Cell lines with activity area within 0.8 SDs of the mean were considered to be intermediate and eliminated from our analysis [9].

### Combining and homogenizing cell line between CCLE and CGP

In order to combine the data generated by two separated laboratories into a uniform model, we implemented an R script *ComBat* [17] from the *sva* library to eliminate batch effects between two expression data sets. Batch effects are subgroups of measurements that have qualitatively different behavior across conditions and are unrelated to the biological or scientific variables in a study. For example, batch effects may occur if a subset of experiments was run on Monday and another set on Tuesday, if two technicians were responsible for different subsets of the experiments, or if two different lots of reagents, chips or instruments were used. *ComBat* used an empirical Bayes method to adjust potential batch effects between two data sets.

### Feature selection by SVM-RFE, F-score and random forest

For many learning domains, a human defines the features that are potentially useful. However, not all of these features may be relevant. In such a case, choosing a subset of the original features will often lead to a better performance. For supervised learning problems including drug sensitivity prediction, feature selection algorithms choose the optimal feature subset through maximizing a function of predictive accuracy.

Three general classes of feature selection algorithms are often used in the literature: filter methods, wrapper methods and embedded methods. F-score is a typical filter method, which applies a statistical measure to assign a scoring to each feature [18, 19]. Features are then ranked by the score and either selected to be kept or removed from the dataset. Given training vectors *χ*_*κ*_, *κ* = 1, …, *m*, if the number of positive and negative instances are *n*_+_ and *n*_−_, respectively, then the F-score of the *i*-th feature is explained as follows:

$$ F(i)=\frac{{\left({\displaystyle \overset{-\left(+\right)}{x_i}}-{\displaystyle \overset{-}{x_i}}\right)}^2+{\left({\displaystyle \overset{-\left(-\right)}{x_i}}-{\displaystyle \overset{-}{x_i}}\right)}^2}{\frac{1}{n_{+}-1}{\displaystyle {\sum}_{k=1}^{n_{+}}{\left({x}_{k,i}^{\left(+\right)}-{\displaystyle \overset{-\left(+\right)}{x_i}}\right)}^2+}\frac{1}{n_{-}-1}{\displaystyle {\sum}_{k=1}^{n_{-}}{\left({x}_{k,i}^{\left(-\right)}-{\displaystyle \overset{-\left(-\right)}{x_i}}\right)}^2}}, $$

where ${\displaystyle \overset{-}{x_i,}}{\displaystyle \overset{-\left(+\right)}{x_i}},{\displaystyle \overset{-\left(-\right)}{x_i}}$ are the average of the *i*-th feature of the whole, positive, and negative data sets, respectively; *x*_*k*,*i*_^(+)^ is the *i*-th feature of the *k*-th positive instance, and *x*_*k*,*i*_^(+)^ is the *i*-th feature of the *k*-th negative instance. The numerator shows the discrimination between the positive and negative sets, and the denominator defines the one within each of the two sets. The larger the F-score is, the more likely this feature is more discriminative. In general, this kind of approaches is easy to implement and computationally efficient, but the drawback is that it considers the feature independently and thus neglects the combination effects between different features.

In our study, features are selected using a recursive feature selection namely SVM-RFE (Support Vector Machine Recursive Feature Elimination). SVM-RFE is a wrapper method by considering feature selection as a search problem, where different combinations are evaluated and compared to other combinations. In detail, it selects optimal features from an initial feature set by the following steps: i) fits a simple linear SVM, ii) ranks the features based on their weights in SVM solution, iii) eliminates the feature with the lowest weight to get the gene rank. Selected top features were then used to fit an SVM model. In contrast to filter-based models, SVM-RFE is computationally expensive, but it is much possibly to find the best feature combination.

The Random Forest (RF) uses a collection of decision tree classifiers, where each tree in the forest has been trained using a bootstrap sample of individuals from the data, and each split attribute in the tree is chosen from among a random subset of attributes. RF is applicable to very high dimensional data with fewer observations and can handle the problems of noisy data and imbalanced classes [20].

### Support vector machine

Support vector machine (SVM) is a supervised learning algorithm that analyzes data and recognizes patterns, used for classification and regression analysis. Basically, the SVM model will represent samples as points in the feature space, such that samples of two categories are divided by a clear gap as wide as possible. New samples are then mapped into the same space and predicted to a category based on which side they fall on.

In addition to linear classification, SVMs can also efficiently perform non-linear classifications using a so-called kernel trick, which implicitly maps the inputs into a higher dimensional feature space. The kernel formulation has two advantages. First, it reduces the number of model parameters to match the number of samples (training cell lines) and not the number of features. Second, it captures nonlinear relationship between genomic and epigenomic features, and cell-line drug sensitivities. In this study, SVM was implemented by the R package *e1071*, where parameters are optimized by a grid search over provided parameter ranges.

### Model based testing

The best number of features and parameters (C and γ) were obtained by minimizing the classification error of SVM based on 10 iterations of 10-fold cross-validation. Different from CCLE, drug sensitivity in CGP was measured by IC_50_ rather than activity area, so the model trained from CCLE is not applicable to CGP directly. But there is a natural relation between activity area and IC_50_, i.e., high activity area corresponds to low IC_50_ as shown in Additional file 1. So we used IC_50_ to classify samples in CGP, while leaving model trained by CCLE to validate this model. For CGP dataset, sample classification is quite similar to that in CCLE, i.e., IC_50_ values for each compound were normalized to zero mean and unit variance. Then, cell lines with IC_50_ at least 0.8 SDs above the mean were defined as resistant, whereas those at least 0.8 SDs below the mean were defined as sensitive. The rest intermediate part is eliminated from our analysis.

When building the model, we selected the optimal parameters by a grid search in the range of cost: {0.1,1,10,100,200,300,500,700,800,1000}, and gamma: {0.1,0.5,1,2,3,4,5,6,7,8}. Next, we evaluated our algorithm by predicting drug responses for an independent dataset CGP using the model trained from CCLE. Finally, *t*-test and ROC curve were explored to assess the robustness of our model.

## Results

### Computational framework

**Fig. 1:**
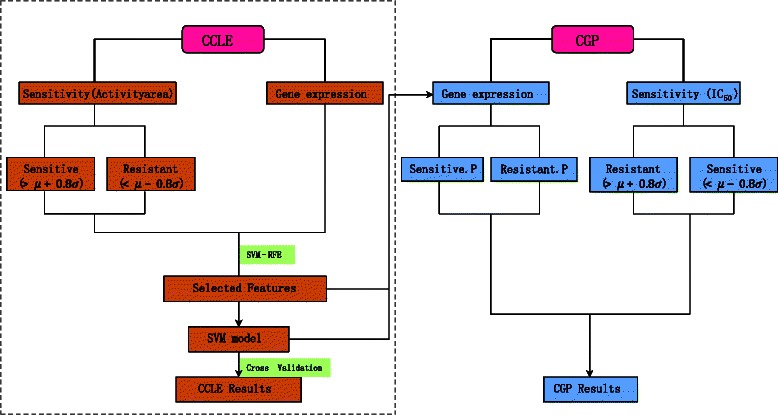
Computational framework. In the left panel, cell lines in CCLE were first divided into three groups according to their normalized drug response values. Then gene expression features were selected by SVM-RFE for building an SVM model, where the optimal feature number and parameters were obtained by a 10-fold cross validation. To test the generalization ability of the model, in the right panel, gene expression profile of CGP data set was fed to the model to get the attribute (sensitive or resistant) of each cell line. Then CGP performance was measured by comparing the model prediction with the sample classification based on the normalized IC_50_

**Fig. 2:**
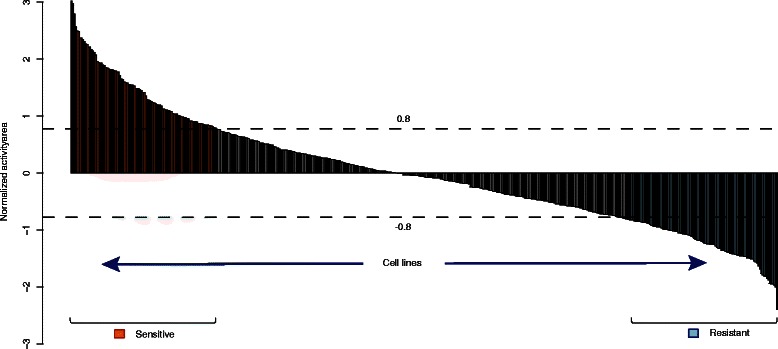
Sample classification for the drug Panobinostat. All samples are classified into three groups according to the threshold 0.8 SDs of the normalized activity area

**Fig. 3:**
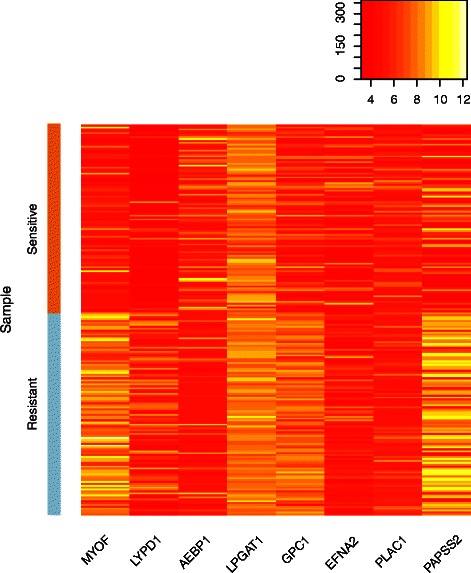
Gene expression of sensitive and resistant cell lines for Panobinostat

The conceptual framework of our study is shown in Fig. 1. In the first step, cell lines in CCLE were divided into three groups (Sensitive, Resistant and Intermediate) according to their normalized sensitivities to a given drug (see Fig. 2 as an example). Samples in sensitive and resistant groups are retained to train an SVM model. After this step, 2 drugs (L-685458 and Nilotinib) ended up with very few valid samples due to the bias of their drug sensitivity distributions, thus were discarded from our further analysis. As is expected, samples in sensitive and resistant groups are shown to have very distinct gene expression patterns (an example in Fig. 3). Next, we used gene expression features selected by SVM-RFE to build an SVM model for the CCLE dataset, where the optimal feature number and parameters were obtained by 10-fold cross validation.

**Fig. 4:**
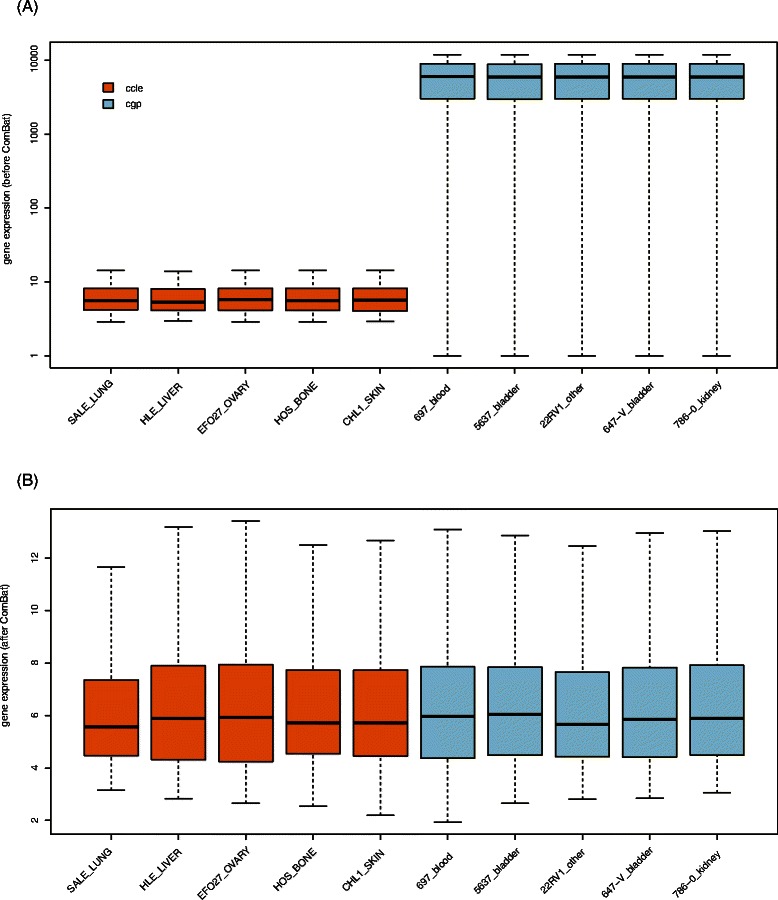
Elimination of Batch effect by ComBat. Boxplot showing gene expression distributions before (**a**) and after (**b**) *ComBat* for five cell lines in CCLE and CGP

As an independent dataset, CGP, was used to further evaluate our method based on the model built from CCLE. Since gene expression profiles of two data sets are conducted by two different platforms and thus have significantly different magnitudes (Fig. 4a), we first removed the batch effects using the *ComBat* function in R (Fig. 4b). Then standardized gene expression profile in CGP was fed to the model built from CCLE to get the attribute (sensitive or resistant) of each cell line. The final result of CGP was got by comparing the predictions with the truth by sample classification based on their IC_50_ values (details are in the Method part).

### Cross validation in CCLE and analysis of selected features cross validation in CCLE

**Fig. 5:**
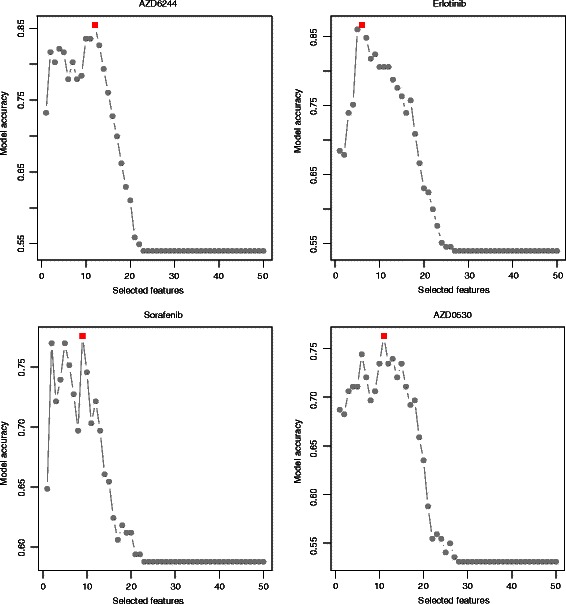
Prediction accuracy and number of selected features for four drugs. Prediction accuracies at different numbers of selected top features for four drugs, i.e., AZD6244, Erlotinib, Sorafenib and AZD0530. The optimal feature numbers are highlighted in red

Our model has three free parameters, i.e., the number of selected top features and two model parameters (*C* and γ) in SVM. Here, a 10-fold cross validation on CCLE dataset is conducted to get the optimal gene features and parameters. Examination on prediction accuracies with respect to numbers of selected features showed a consistent trend of increasing first and decreasing afterwards with the increase of selected features (see four examples in Fig. 5). We concluded that, for all drugs tested, only a few genes could be enough to enable a satisfactory accuracy. The optimal gene numbers and parameters for drugs in CCLE are listed in Additional file 2.

**Fig. 6:**
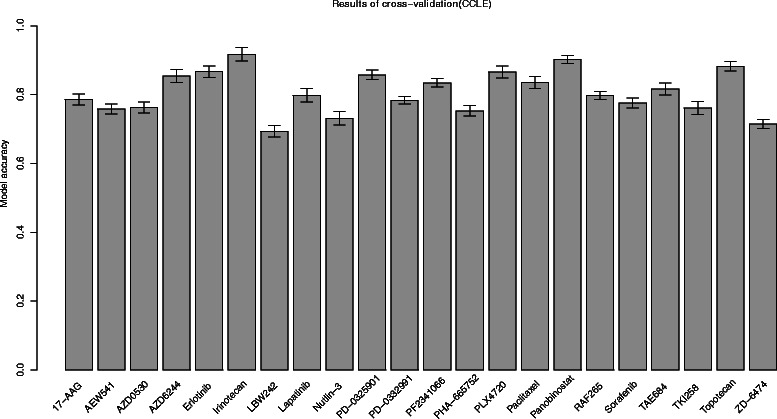
Cross validation results for CCLE drugs. For each drug in CCLE, model accuracy was obtained through a 10-fold cross validation. Barplot shows accuracy values for all drugs in CCLE

Next, an SVM model was built for each drug after getting the optimal features and model parameters conducted by 10-fold cross validation (Fig. 6). By 10-fold cross validation, accuracies of our model are around 80 % for most drugs in CCLE, and the highest accuracy of 91.73 % was attained for a pathway targeted compound, the topoisomerase 1 inhibitor Irinotecan. The kind of phenomenon was also reported by Jang et al., who showed that pathway targeted compounds lead to more accurate predictors than classical broadly cytotoxic chemotherapies [21]. Performance of two MEK inhibitors (AZD6244, PD-0325901) was also quite promising with the model accuracies of 85.44 % and 85.78 %, respectively. Accuracies for four EGFR inhibitors are 76.3 %, 86.67 %, 79.77 % and 76.17 %, respectively. The lowest accuracy of 69.35 % was obtained for LBW242, which is also the worst prediction in the CCLE paper [22], implying the consistence of our result with the Elastic net model.

To further emphasize the fact that drug response can be predicted from genomic features, we clustered all cell lines in CCLE dataset based on their baseline gene expressions (Additional file 3). Then examined whether there are significant differences between these clusters in terms of copy number variant or mutation status. Results indicate that there are significant differences in copy number and mutation status between different clustering categories (Additional file 4).

### Selected features are associated with tumorigenesis or drug response

Selected genes for CCLE drugs are shown in Additional file 2 and their functions in tumorigenesis are listed in Additional file 5. It is shown that many selected genes are reported to have close relationship with tumorigenesis or cancer progression. For example, the selected top features for AZD6244 are SPRY2, FAM127B, GDF15, CAST, DAB2, CLEC11A, PRRG1, EDN1, CCL20, AXL, PPAP2C and ITGA4. Among them, SPRY2 is reported to have a consistent repressive expression in malignant hepatocytes compared with normal or cirrhotic hepatocytes in human hepatocellular carcinoma where the MAPK activity is enhanced via multiple hepatocarcinogenic factors [23]. GDF15 was also reported as an epigenetic biomarker for detection of bladder cancer from DNA-Based analyses of Urine samples [24]. In a recent study of microarray-based methylated-CpG island recovery assay, hypermethylation and low expression level of ITGA4 were reported to be enriched in breast cancers [25]. Direct bisulfite sequencing also showed widespread methylation occurring in intragenic regions of the WT1, PAX6 and ITGA4 genes and in the promoter region of the OTX2 gene in breast cancer tissues [25].

In order to test the effectiveness of SVM-RFE, feature selection was also conducted by F-score [18, 19] and random forest [26–28]. Results indicate that model based on SVM-RFE (≥80 % accuracy for 10 drugs, ≥ 75 % accuracy for 19 drugs) achieves much better performance than F-score (≥80 % accuracy for 1 drugs, ≥ 75 % accuracy for 5 drugs) for all drugs and random forest (≥80 % accuracy for 8 drugs, ≥ 75 % accuracy for 10 drugs, Additional file 6). Furthermore, random forest was used to predict drug sensitivity (CGP IC_50_). Results reveal that SVM prediction model achieves better performance for some drugs (Erlotinib, Paclitaxel and PF-2341066 etc.) than random forest model (Detailed results in Additional files 7,8, 9, 10).

### Independent validation in CGP

Next, we further validated our algorithm by an independent dataset CGP based on the model trained from CCLE. Since CCLE and CGP were generated by two different consortiums and platforms, the total numbers of genes and expression distributions are significantly different between these two data sets. To make sure a uniform data distribution, the *ComBat* function from the *sva* package in R is applied to these two data sets to remove the batch effect.

**Fig. 7:**
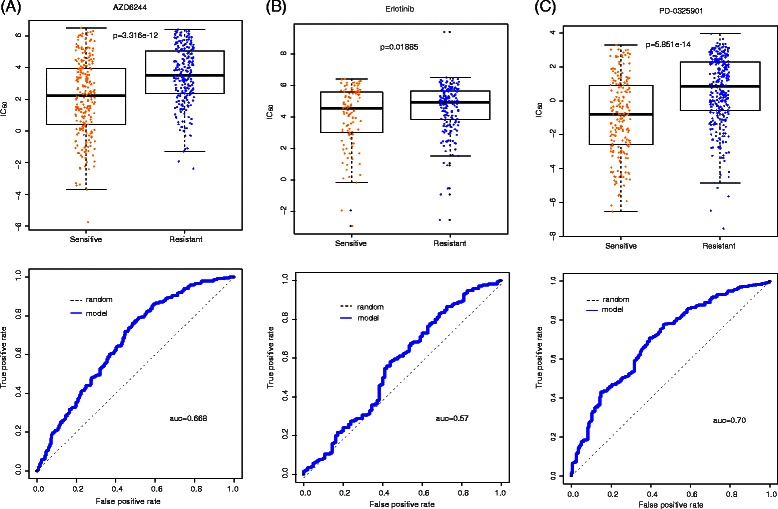
Independent tests on CCLE model for AZD6244, Erlotinib and PD-0325901. Boxplot and ROC curve (the bottom curve indicates drug response, measured as the area over the dose–response curve, i.e., activity area) have been built to evaluate the svm model. (**a**) For drug AZD6244, p-value by *t* test is 3.316e-12 and area under the curve is 0.668. (**b**) For drug Erlotinib, p-value by *T* test is 0.01885 and area under the curve is 0.57. (**c**) For drug PD-0325901, p-value by *t* test is 5.851e-14 and area under the curve is 0.70

Performances of 11 common drugs between CCLE and CGP are shown in Additional file 2. As is shown, 3 of these 11 drugs (AZD6244, Erlotinib and PD-0325901) achieve a relatively good performance of AUC from 0.57 to 0.7 (Fig. 7), but the rest eight drugs only give the AUC values around 0.5 (Additional files 11 and 12). Predicted drug responses of sensitive and resistant samples are significantly different for AZD6244 (Fig. 7a, *p*-value = 3.316e-12 by t.test), PD-0325901 (*p*-value = 5.851e-14) and Erlotinib (*p*-value = 1.885e-2).

In addition, we also built an SVM model for each drug in CGP using IC_50_ as drug response measurement according to the same procedure, and got the gene rank list according to their importance (termed as “CGP_Impor”) in drug sensitivity prediction (Additional file 13). In order to test the consistency of this list with that by CCLE (termed as “CCLE_Impor”), we split these two lists (top 1500 genes) into 3 groups and examined their overlaps in each group (Table 1). Results by Fisher’s exact test indicate that the overlap between CGP_Impor and CCLE_Impor are significantly.

**Table 1:** Overlap between selected top features from CCLE and CGP. For each drug, the table shows the number of common genes between CCLE_Impor and CGP_Impor and overlapping significance by Fisher’s exact test

Drug	1-500	*p*-value	501-1000	*p*-value	1001-1500	*p*-value
AZD6244	79	3.716	49	3.277	30	3.48
		e-25		e-8		e-2
Erlotinib	79	3.716	47	2.225	38	3.47
		e-25		e-7		e-4
PHA665752	72	1.603	40	8.163	33	7.76
		e-20		e-5		e-3
AZD0530	72	1.603	42	1.722	32	1.32
		e-20		e-5		e-2
Paclitaxel	74	8.309	45	1.368	39	1.71
		e-22		e-6		e-4
PF-2341066	80	7.541	41	3.801	28	8.06
		e-26		e-5		e-2
Sorafenib	79	3.716	31	2.177	22	0.46
		e-25		e-2		
PD-0325901	78	1.80	54	1.803	28	8.06
		e-24		e-10		e-2
Lapatinib	68	4.78	46	5.586	33	7.76
		e-18		e-7		e-3
PLX4720	78	1.80	41	3.801	29	5.38
		e-24		e-5		e-2
PD-0332991	68	4.78	39	1.706	28	8.06
		e-18		e-4		e-2

## Discussion and Conclusions

The generation of genetic predictions of drug response in the preclinical setting and their incorporation into cancer clinical trial design could speed the emergence of “personalized” therapeutic regimens. In our study, a robust predictor was built for this purpose using an SVM model after recursive feature selection. 10-fold cross validation on CCLE data set showed that our model achieves the accuracy of over 80 % for 10 of 22 drugs. Independent test on CGP suggests that only 3 of 11 common drugs between CCLE and CGP get satisfying result, further implying the inconsistency between these two data sets. The novelty of our algorithm lies on the following aspects. First, most previous work on drug response prediction mainly based on individual dataset, such as NCI60, CCLE or CGP, but seldom see integration analysis. We combined datasets generated by two important studies and further checked their consistency in drug response profiles. Second, a backward feature selection approach based on linear-kernel SVM was used to selected drug response-relevant features instead of a screening scheme by CCLE and CGP. So combination effects of features could be possibly captured by our model compared to filter methods such as F-score. Finally, we transformed the original regression problem into a classification problem by a discretization strategy, thus more machine-learning tools could be incorporated to this problem.

Since mutation and copy number variation information are also important indicators for drug response and available in CCLE and CGP studies, we further investigated whether a joint model by integrating these information could possibly improve drug response prediction. So we combined gene expression, copy number and gene mutation data sets into an integrated dataset, and conducted SVM-RFE for feature selection based on the integrated dataset. Comparative results showed that the integrated model achieved only slightly higher prediction accuracies for most drugs in CCLE (Additional files 2 and 14), indicating the central role of gene expression in drug response prediction. Similar phenomenon was also observed in a recent comparison study by Costello et al., who concluded that gene expression data provides the most predictive power for any individual profiling data set [29]. So for the sake of generalization capability of our model, it is much practical to use only gene expression to construct prediction model rather than all genomic features.

However, our model also suffered from the following limitations that can be addressed in our future work. First, besides gene expression, epigenetic and protein level information also play very important roles in drug response mechanism, and thus should be incorporated in the prediction model. Second, in our model, expressions of different genes are assumed to be independent with each other, but it is not the truth since functionally related genes could form a pathway or molecular complex to execute a specific biological process. So further attention should be paid on taking these functional structures into consideration for a better prediction of drug response.

## Additional files

Additional file 1: **Relationships between activity area and IC**_**50**_**of drug AEW541.** For drug AEW541, a scatterplot was drawn to reveal the relationship between activity area and IC_50_ with a p-value by spearman correlation.
Additional file 2: **Relevant information of CCLE drugs.** In this study, we analyzed 22 drugs in CCLE, here we listed the relevant information of these drugs including model results and selected features that derive from different data sets (EXP vs EXP + CPV + SNP). Also, feature selection was conducted by F-score and random forest, then selected features were used to build SVM model (Relevant information can be seen here).
Additional file 3: **Results of Consensus Cluster in gene expression dataset.** All cell lines in CCLE dataset were clustered based on their baseline gene expression. **(A)** Results when gene expression dataset were divided into four categories. **(B, C)** In the process of Consensus cluster, relative change in area under CDF curve tend to be stable when k = 4. Then this value provides us with a basis for classification.
Additional file 4: **Results of p.values returned from t.test and Fisher’s exact test.** To further emphasize the fact that drug response can be predicted from genomic features, all cell lines in CCLE dataset were clustered based on their baseline gene expression. Then t.test and Fisher’s exact test are used to examine whether there are significant differences between these clusters in terms of copy number variant and mutation status, respectively. Results indicate that differences do exist between different categories of cpv and snp data sets.
Additional file 5: **Relationships between selected features and cancer.** For drug AZD6244, Erlotinib and PD-0325901, functions of selected genes in tumorigenesis are listed here. Many selected genes are reported to have close relationship with tumorigenesis or cancer progression. (**A**) Relationships between selected features and cancer for drug AZD6244. (**B**) Relationships between selected features and cancer for drug Erlotinib. (**C**) Relationships between selected features and cancer for drug PD-0325901.
Additional file 6: **Results of cross validation based on different feature selection methods (SVM-RFE, F.score and Random Forest).** Feature selection was also performed by means of F.score and Random Forest in order to demonstrate the efficiency of SVM-RFE. Then the selected features were used to build the SVM model. Also, 10-fold cross validation was conducted to test the robustness of the model. Comparison of the model accuracy showed that features returned from SVM-RFE have better generalization ability.
Additional file 7: **Independent tests for AZD0530, AZD6244 and Erlotinib in random forest predicting model.** Boxplot and ROC curve (the bottom curve indicates drug response, measured as the area over the dose–response curve, i.e., activity area) have been built to evaluate the model. (A) For drug AZD0530, p-value by *t* test is 1.609e-4 and area under the curve is 0.626. (B) For drug AZD6244, p-value by *t* test is 3.45e-13 and area under the curve is 0.715. (C) For drug Erlotinib, p-value by *t* test is 0.42882 and area under the curve is 0.526.
Additional file 8: **Independent tests for Lapatinib, Nutlin-3 and PD-0325901 in random forest predicting model.** Boxplot and ROC curve (the bottom curve indicates drug response, measured as the area over the dose–response curve, i.e., activity area) have been built to evaluate the model. (A) For drug Lapatinib, p-value by *t* test is 2.864e-4 and area under the curve is 0.619. (B) For drug Nutlin-3, p-value by *t* test is 0.04791 and area under the curve is 0.636. (C) For drug PD-0325901, p-value by *t* test is 2.2e-16 and area under the curve is 0.773.
Additional file 9: **Independent tests for PD-0332991, PF-2341066 and PHA-665752 in random forest predicting model.** Boxplot and ROC curve (the bottom curve indicates drug response, measured as the area over the dose–response curve, i.e., activity area) have been built to evaluate the model. (A) For drug PD-0332991, p-value by *t* test is 0.6806 and area under the curve is 0.539. (B) For drug PF-2341066, p-value by *t* test is 0.1792 and area under the curve is 0.506. (C) For drug PHA-665752, p-value by *t* test is 4.231e-3 and area under the curve is 0.391.
Additional file 10: **Independent tests for PLX4720, Paclitaxel and Sorafenib in random forest predicting model.** Boxplot and ROC curve (the bottom curve indicates drug response, measured as the area over the dose–response curve, i.e., activity area) have been built to evaluate the model. (A) For drug PLX4720, p-value by *t* test is 1.429e-7 and area under the curve is 0.609. (B) For drug Paclitaxel, p-value by *t* test is 0.5993 and area under the curve is 0.482. (C) For drug Sorafenib, p-value by *t* test is 0.9058 and area under the curve is 0.488.
Additional file 11: **Independent tests on CCLE model for PHA-665752, AZD0530, Paclitaxel and Sorafenib.** The above graph (boxplot, roc curve) shows the performance of 4 drugs—PHA-665752, AZD0530, Paclitaxel and Sorafenib.
Additional file 12: **Independent tests on CCLE model for PD-0332991, PLX4720, Lapatinib, and PF-2341066.** The above graph (boxplot, roc curve) shows the performance of 4 drugs—PD-0332991, PLX4720, Lapatinib and PF-2341066.
Additional file 13: **Independent tests on CGP model for AZD6244, Erlotinib, PD-0325901.** CGP IC_50_ data was used to build svm model, and then CCLE activity area data was used to test the model. Boxplot and ROC curve have been built to evaluate the svm model. For drug AZD6244, p-value by *t* test is 7.717e-4 and area under the curve is 0.621. For drug Erlotinib, p-value by *t* test is 0.03194 and area under the curve is 0.555. For drug PD-0325901, p-value by *t* test is 1.487e-10 and area under the curve is 0.678.
Additional file 14: **Results of cross validation between different data sets (EXP vs EXP + CPV + SNP).** Gene expression, copy number and gene mutation data sets were combined into an integrated data sets. Then this integrated data sets was used to conduct SVM-RFE and feature selection. Consequently selected features were used to build SVM model. For each drug in CCLE, 10-fold cross validation was performed to test the robustness of the model. Comparison of cross validation results between different data sets (EXP vs EXP + CPV + SNP) can be seen in this barplot.

## Abbreviations

CCLE: The cancer cell line encyclopedia
CGP: The cancer genome project
SVM: Support vector machine
RFE: Recursive feature selection
CCLE_Impor: The gene rank list according to their importance in drug sensitivity prediction when SVM model was built in CCLE using activity area as drug response measurement
CGP_Impor: The gene rank list according to their importance in drug sensitivity prediction when SVM model was built in CGP using IC_50_ as drug response measurement
