# Supplementary figures and images for: Anticancer drug sensitivity prediction in cell lines from baseline gene expression through recursive feature selection
Source: BMC Cancer. 2015 Jun 30;15:489. doi: 10.1186/s12885-015-1492-6 (PMC4485860; doi:10.1186/s12885-015-1492-6)

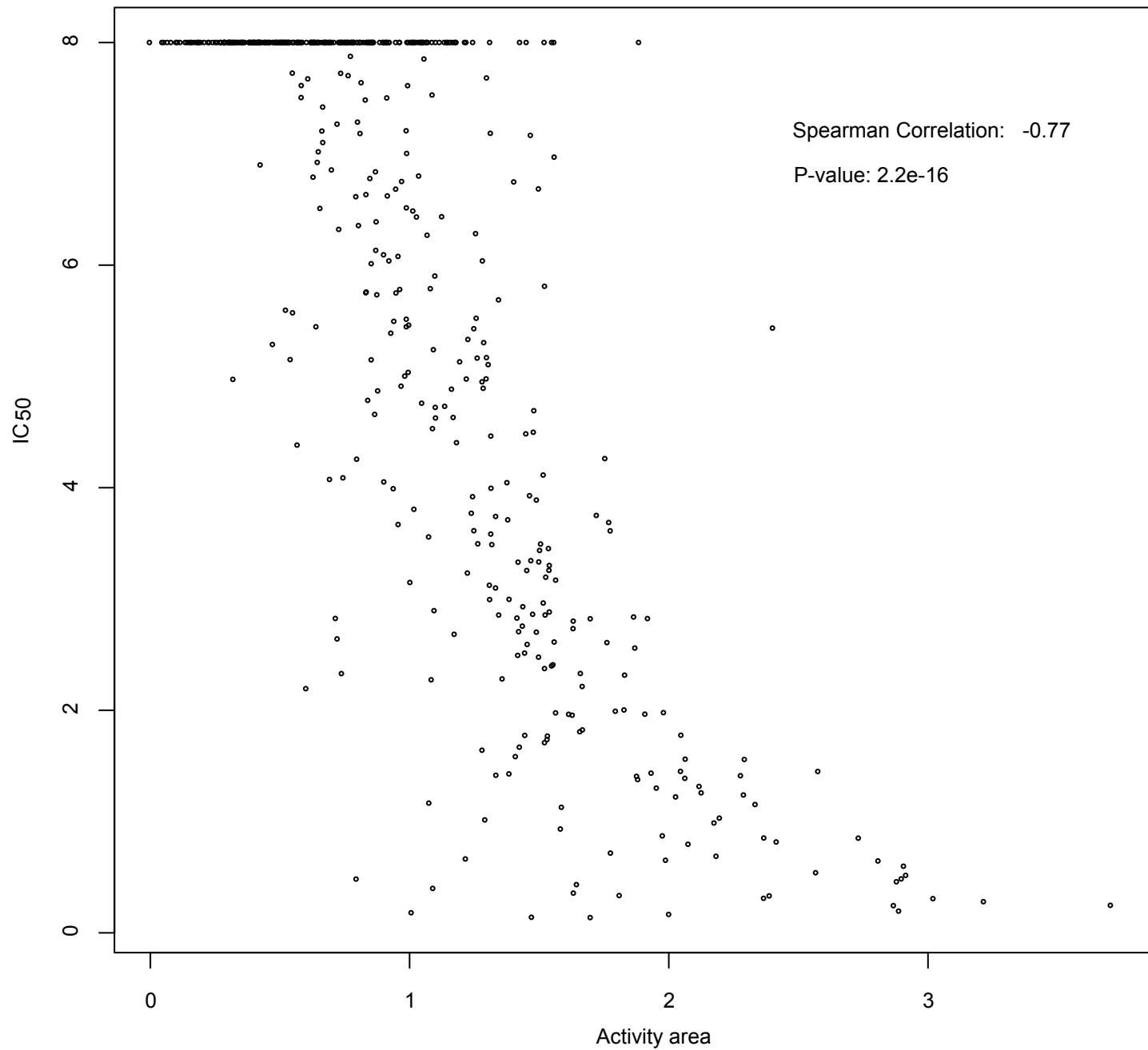

Supplement: Additional file 1: — Relationships between activity area and IC50of drug AEW541. For drug AEW541, a scatterplot was drawn to reveal the relationship between activity area and IC50 with a p-value by spearman correlation. [file 12885_2015_1492_MOESM1_ESM.pdf]

(A)

sample

consensus matrix k=4

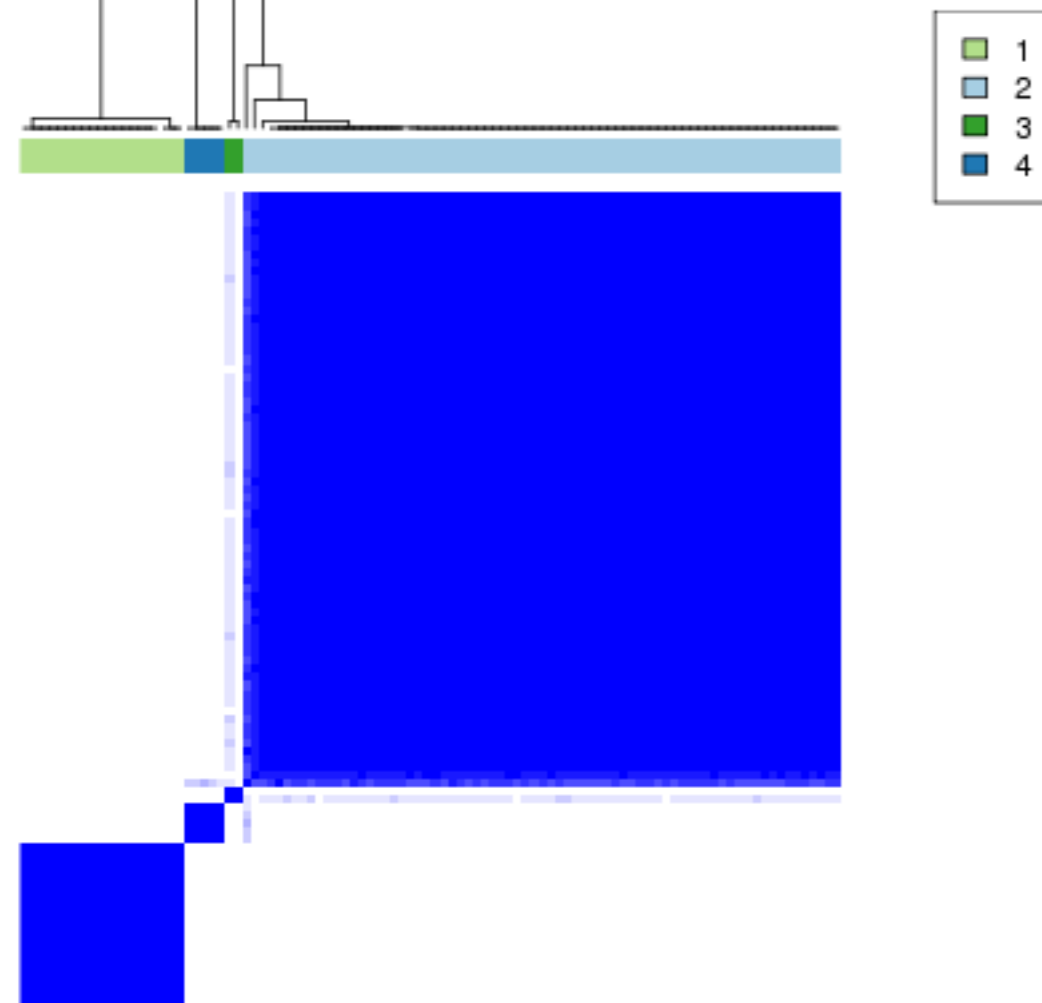

sample

(B)

consensus CDF

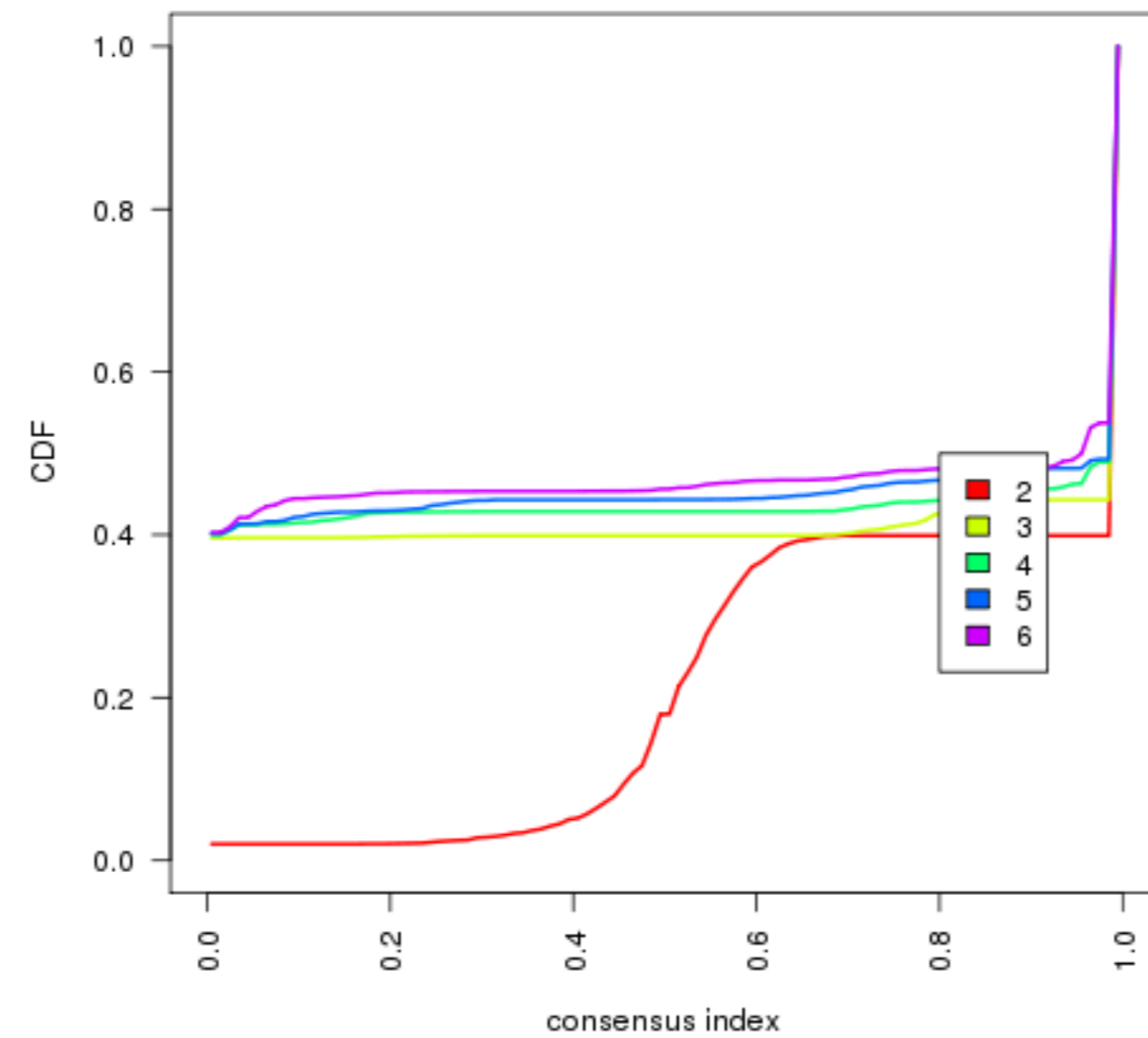

(C)

Delta area

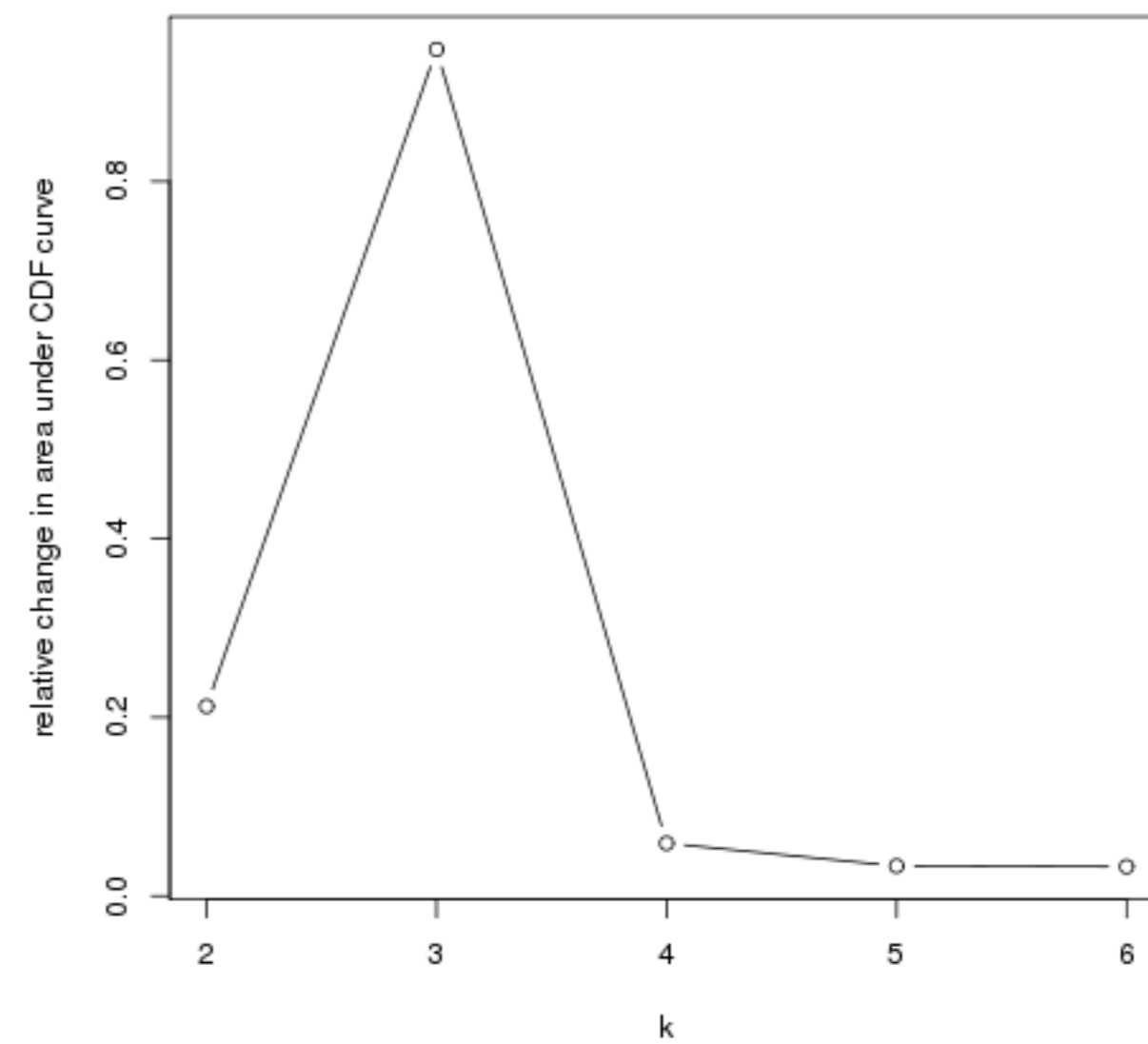

Supplement: Additional file 3: — Results of Consensus Cluster in gene expression dataset. All cell lines in CCLE dataset were clustered based on their baseline gene expression. (A) Results when gene expression dataset were divided into four categories. (B, C) In the process of Consensus cluster, relative change in area under CDF curve tend to be stable when k = 4. Then this value provides us with a basis for classification. [file 12885_2015_1492_MOESM3_ESM.pdf]

Results of cross-validation based on different feature selection methods

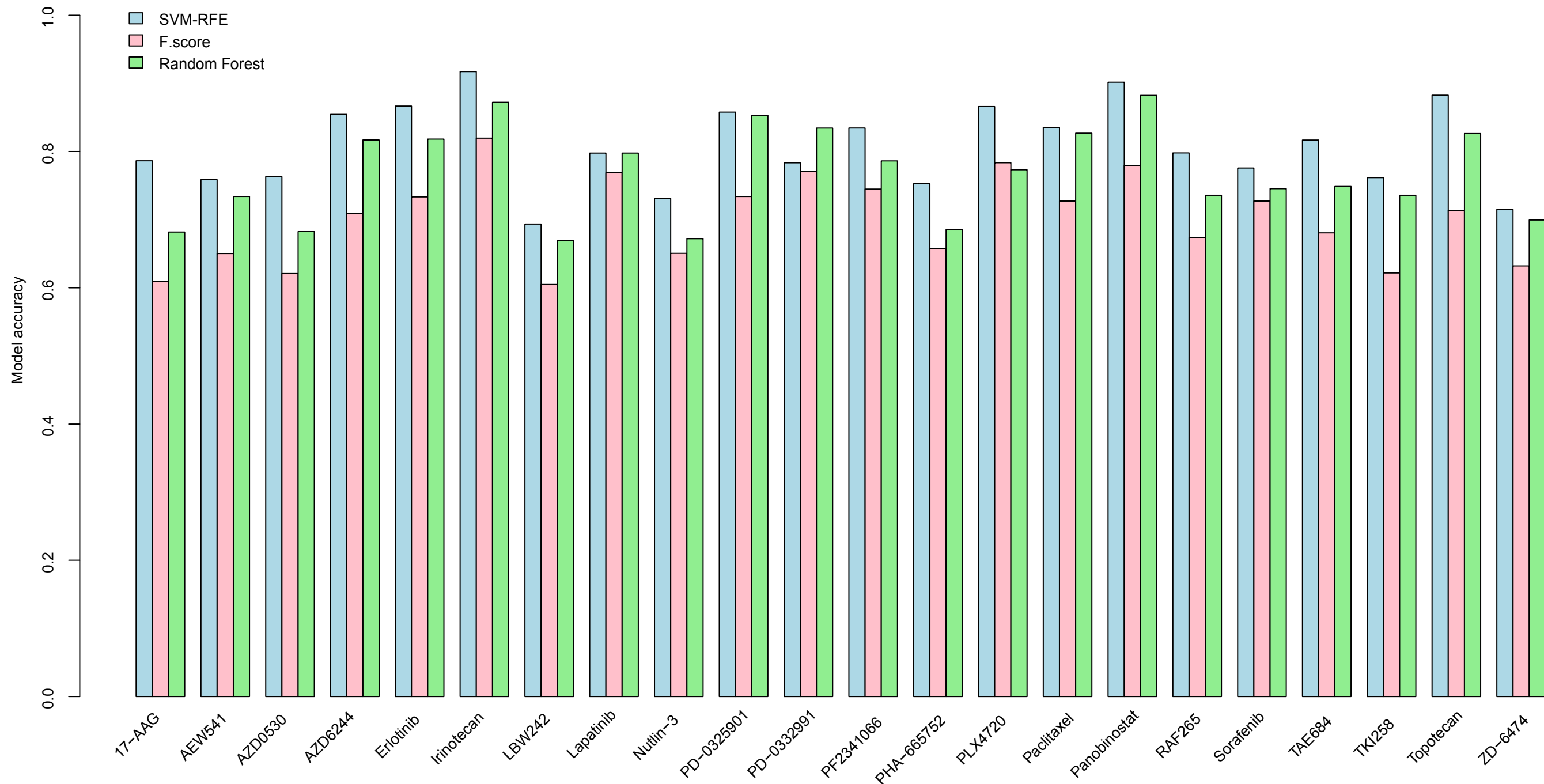

Supplement: Additional file 6: — Results of cross validation based on different feature selection methods (SVM-RFE, F.score and Random Forest). Feature selection was also performed by means of F.score and Random Forest in order to demonstrate the efficiency of SVM-RFE. Then the selected features were used to build the SVM model. Also, 10-fold cross validation was conducted to test the robustness of the model. Comparison of the model accuracy showed that features returned from SVM-RFE have better generalization ability. [file 12885_2015_1492_MOESM6_ESM.pdf]

AZD0530

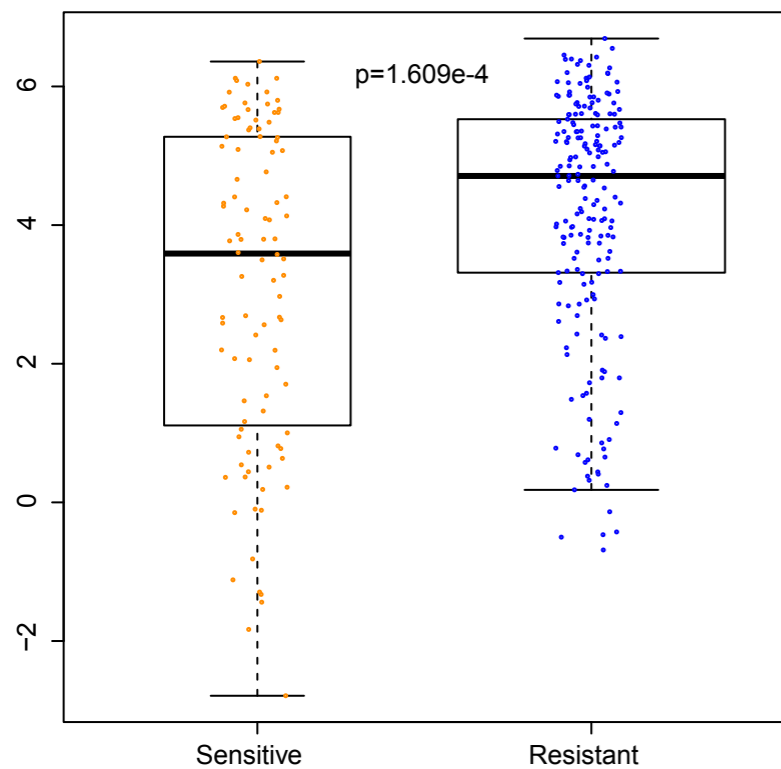

AZD6244

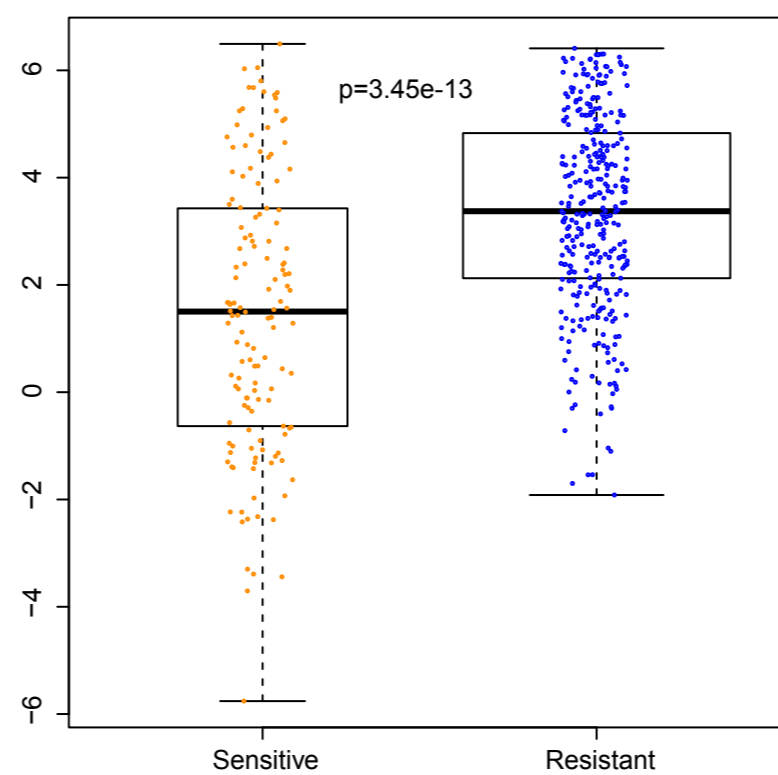

Erlotinib

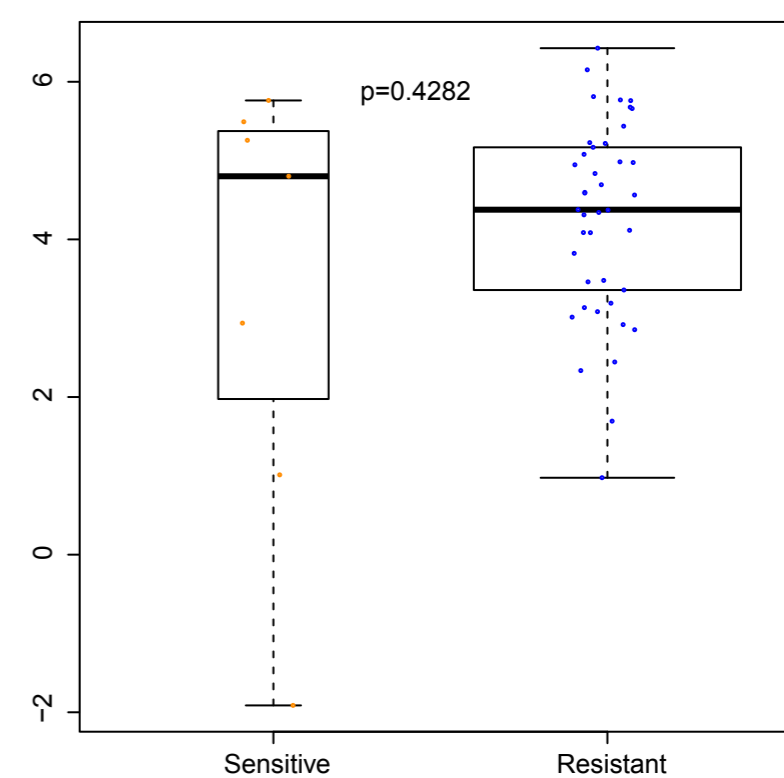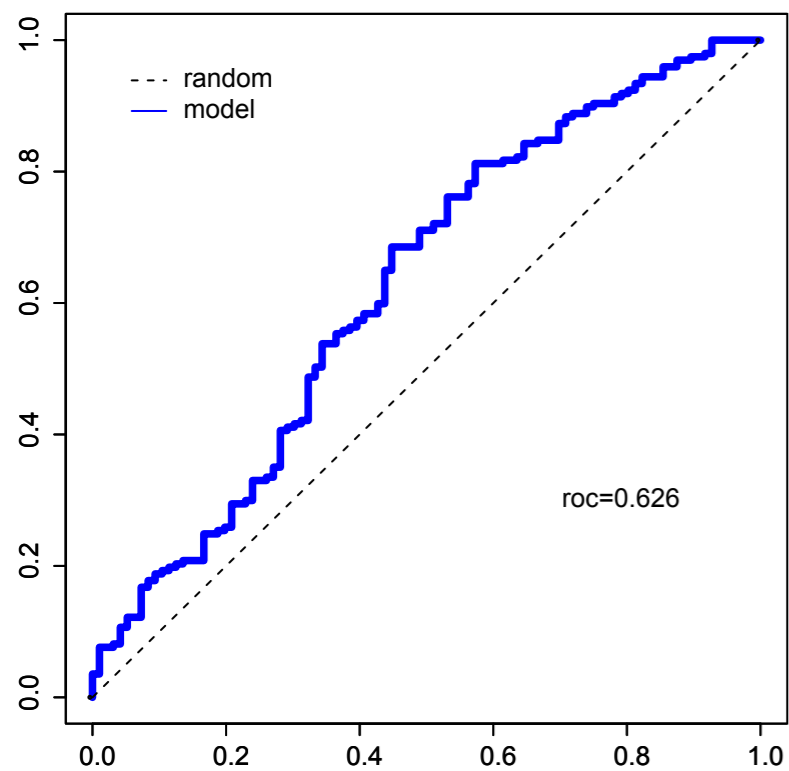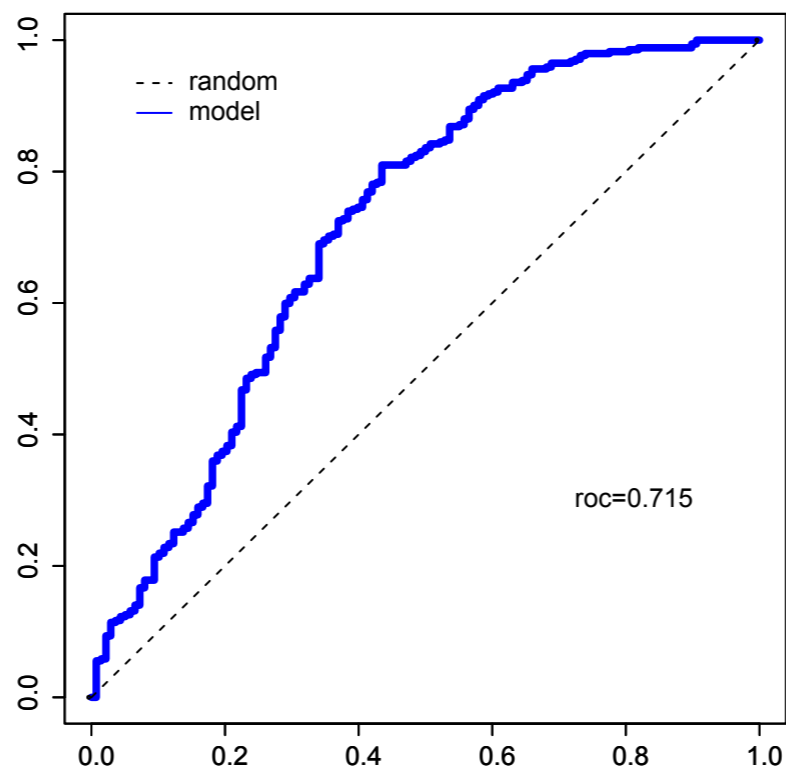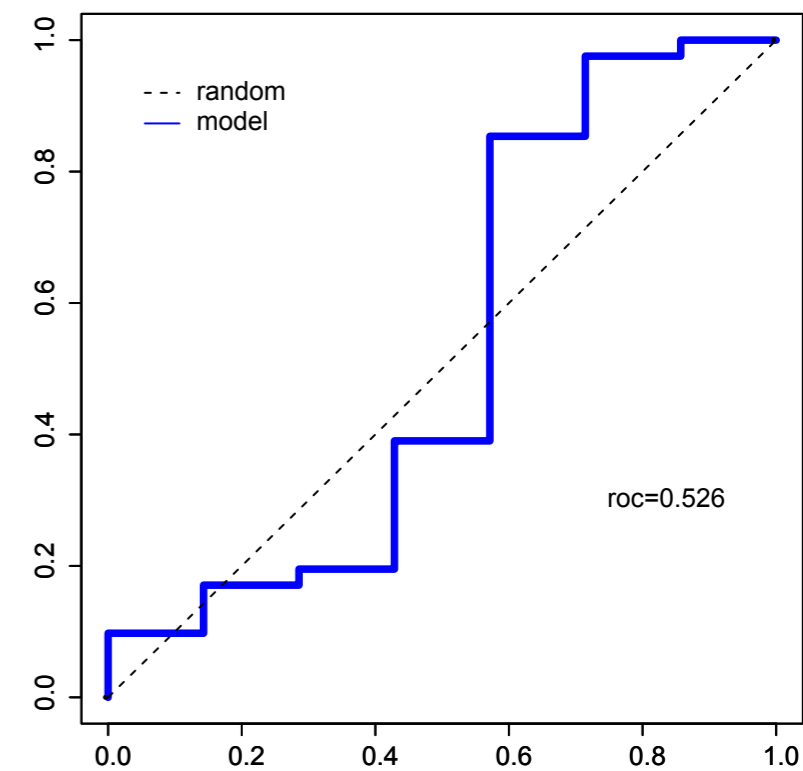

Supplement: Additional file 7: — Independent tests for AZD0530, AZD6244 and Erlotinib in random forest predicting model. Boxplot and ROC curve (the bottom curve indicates drug response, measured as the area over the dose–response curve, i.e., activity area) have been built to evaluate the model. (A) For drug AZD0530, p-value by t test is 1.609e-4 and area under the curve is 0.626. (B) For drug AZD6244, p-value by t test is 3.45e-13 and area under the curve is 0.715. (C) For drug Erlotinib, p-value by t test is 0.42882 and area under the curve is 0.526. [file 12885_2015_1492_MOESM7_ESM.pdf]

Lapatinib

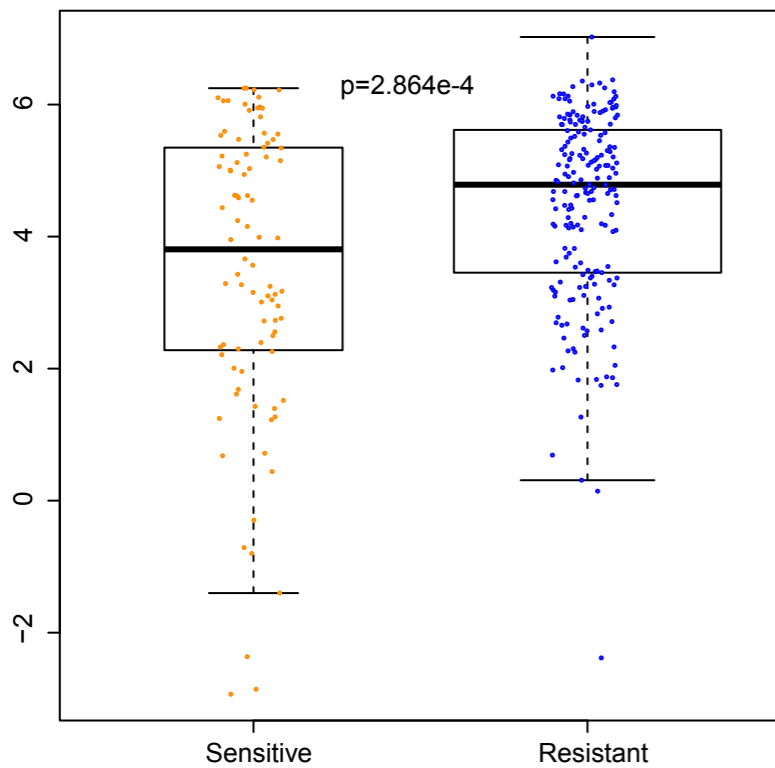

Nutlin-3

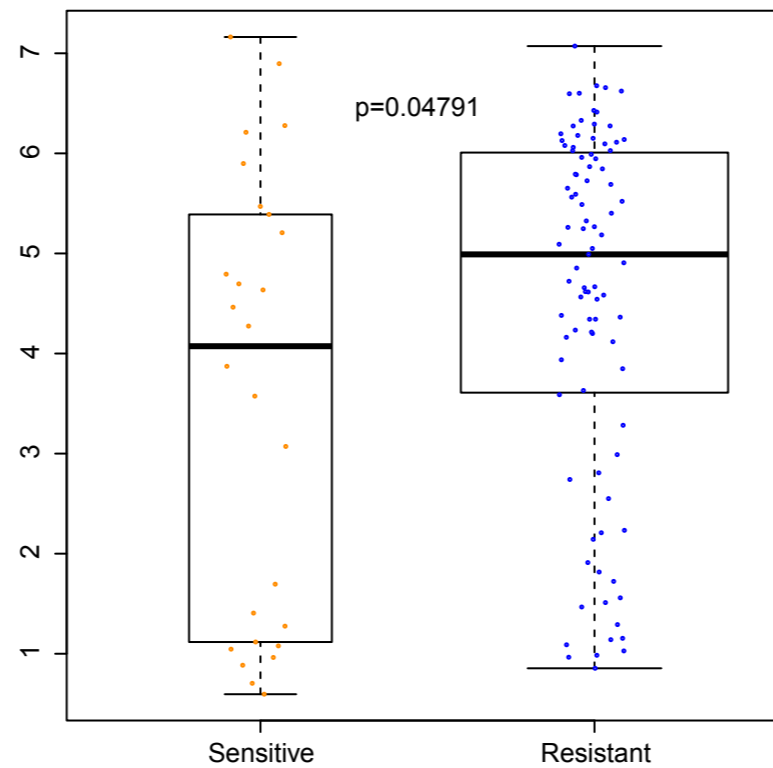

PD-0325901

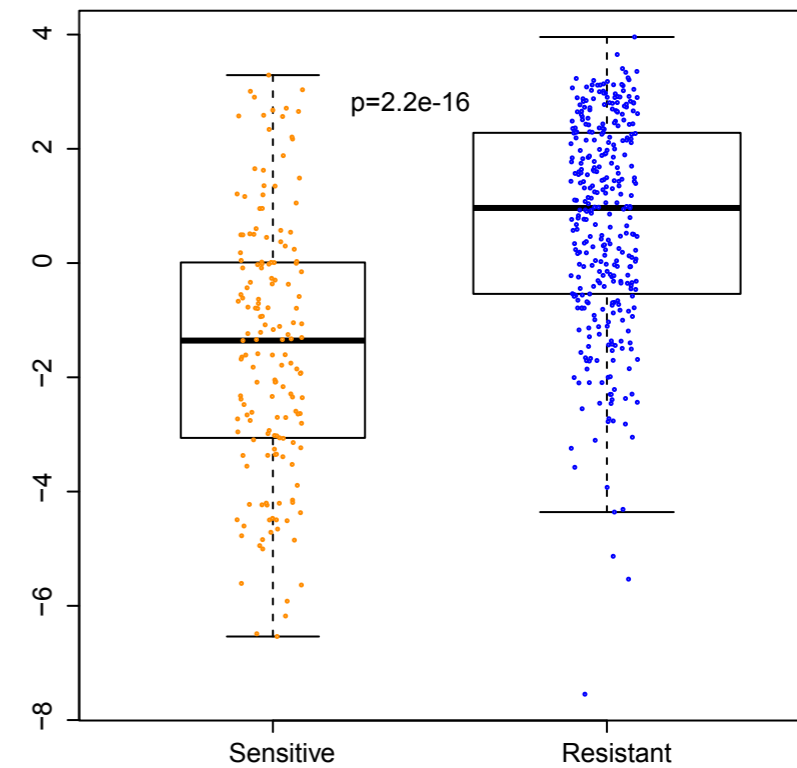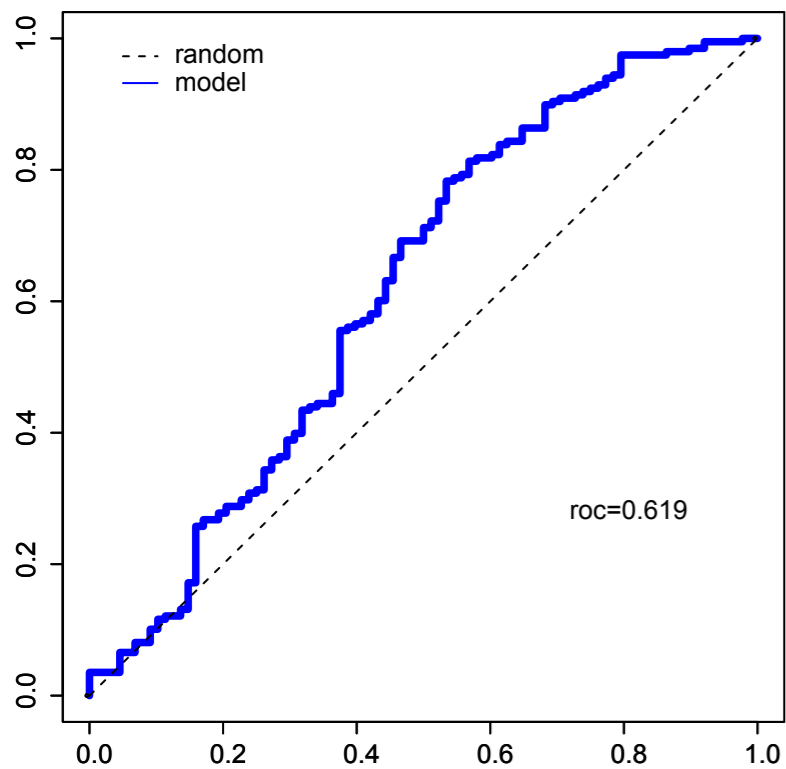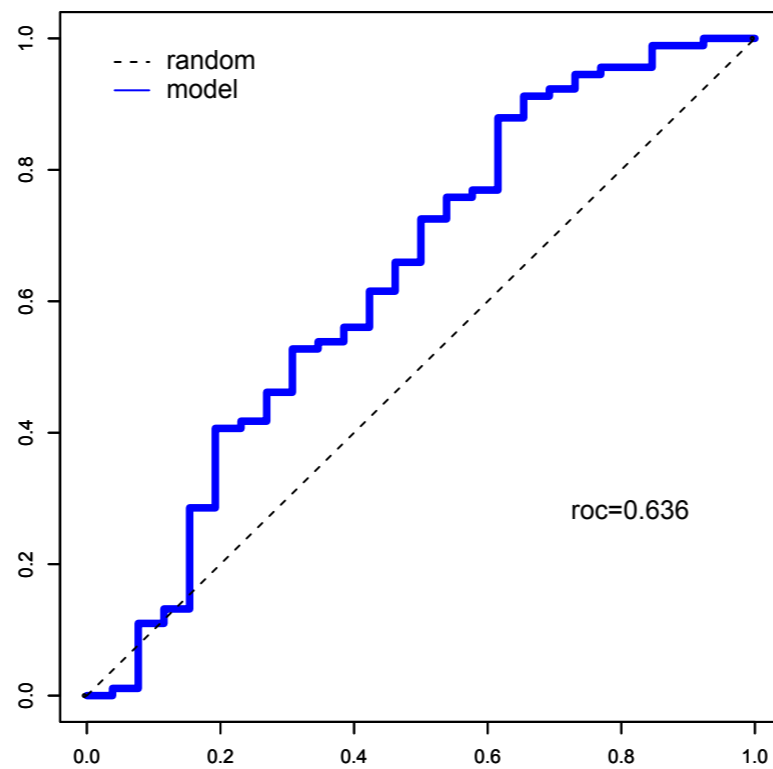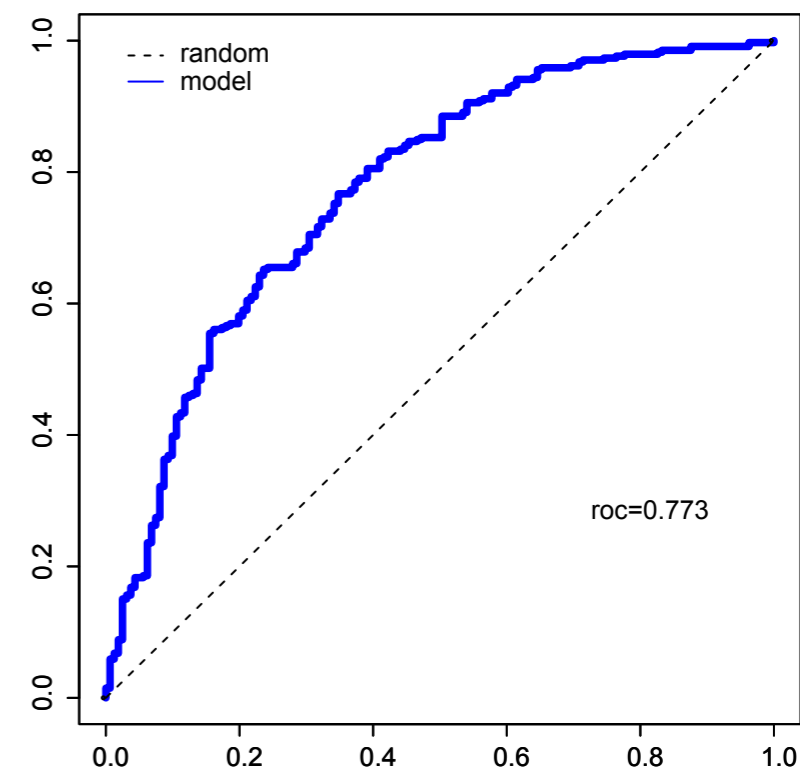

Supplement: Additional file 8: — Independent tests for Lapatinib, Nutlin-3 and PD-0325901 in random forest predicting model. Boxplot and ROC curve (the bottom curve indicates drug response, measured as the area over the dose–response curve, i.e., activity area) have been built to evaluate the model. (A) For drug Lapatinib, p-value by t test is 2.864e-4 and area under the curve is 0.619. (B) For drug Nutlin-3, p-value by t test is 0.04791 and area under the curve is 0.636. (C) For drug PD-0325901, p-value by t test is 2.2e-16 and area under the curve is 0.773. [file 12885_2015_1492_MOESM8_ESM.pdf]

PD-0332991

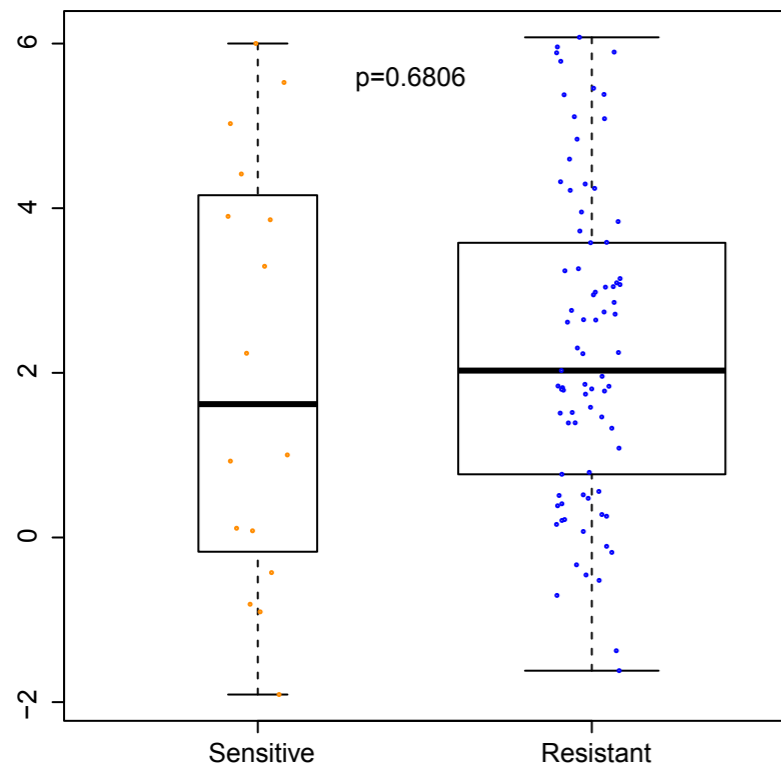

PF-2341066

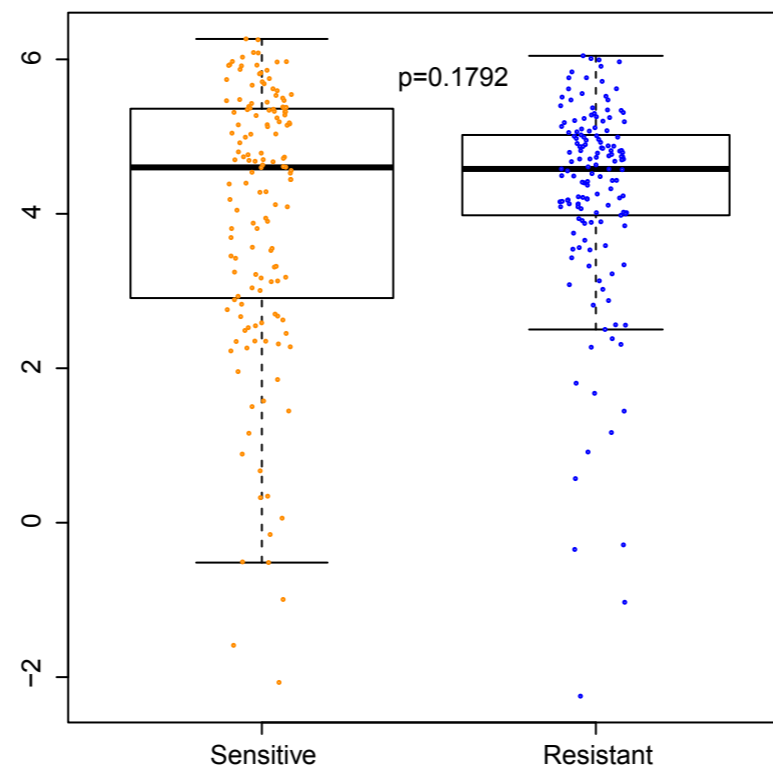

PHA-665752

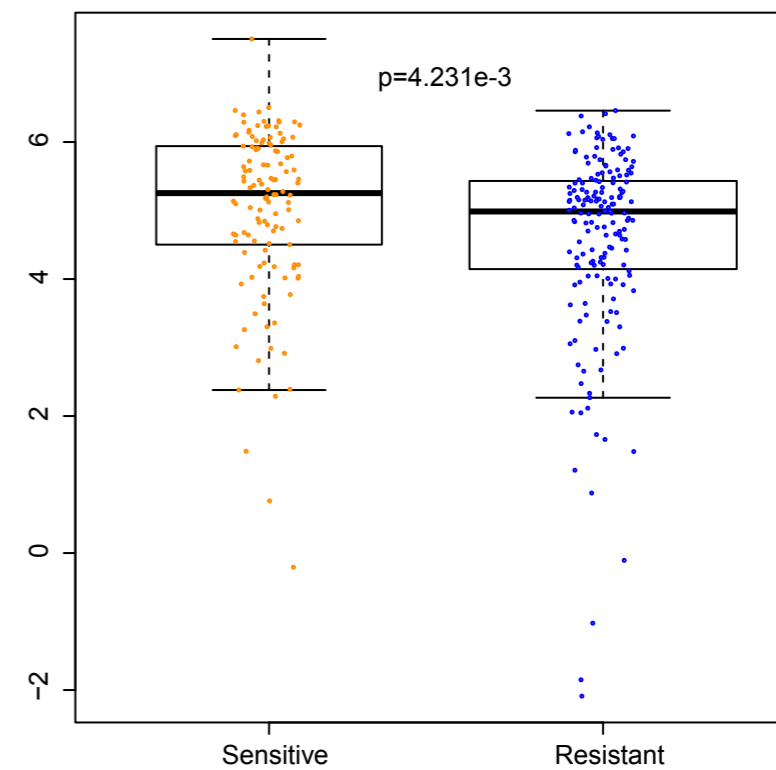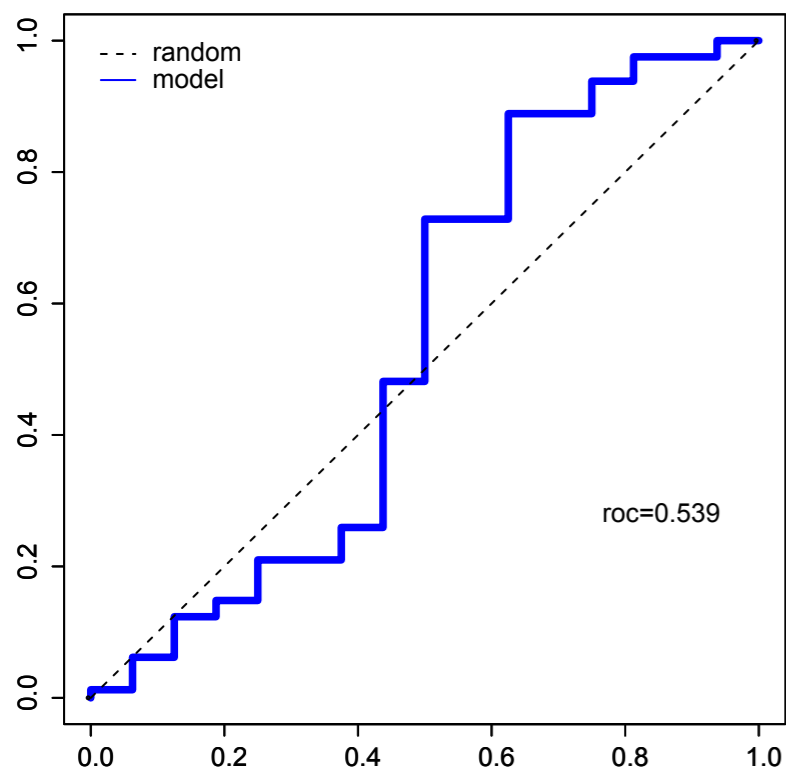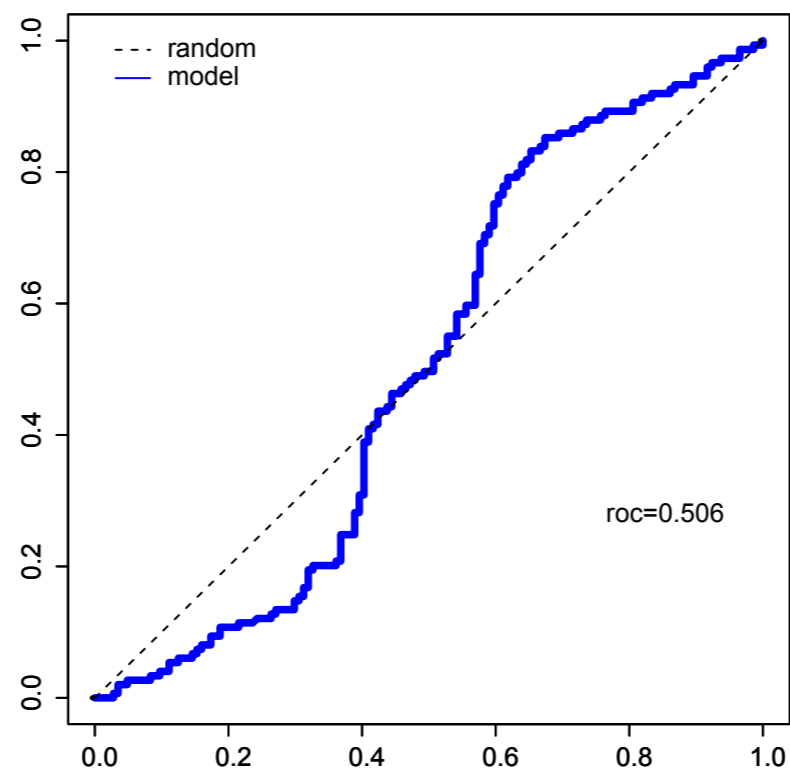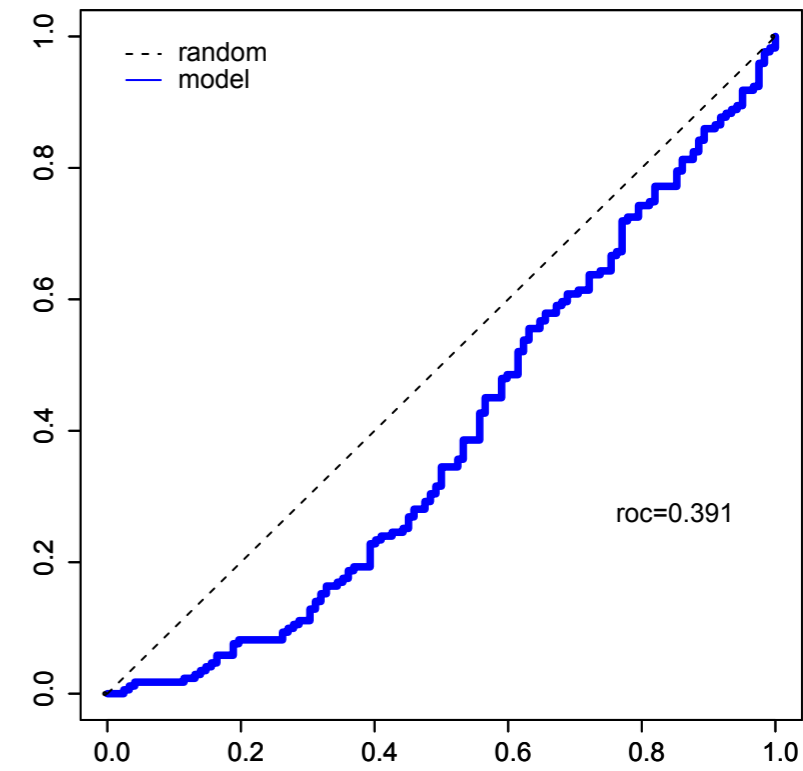

Supplement: Additional file 9: — Independent tests for PD-0332991, PF-2341066 and PHA-665752 in random forest predicting model. Boxplot and ROC curve (the bottom curve indicates drug response, measured as the area over the dose–response curve, i.e., activity area) have been built to evaluate the model. (A) For drug PD-0332991, p-value by t test is 0.6806 and area under the curve is 0.539. (B) For drug PF-2341066, p-value by t test is 0.1792 and area under the curve is 0.506. (C) For drug PHA-665752, p-value by t test is 4.231e-3 and area under the curve is 0.391. [file 12885_2015_1492_MOESM9_ESM.pdf]

PLX4720

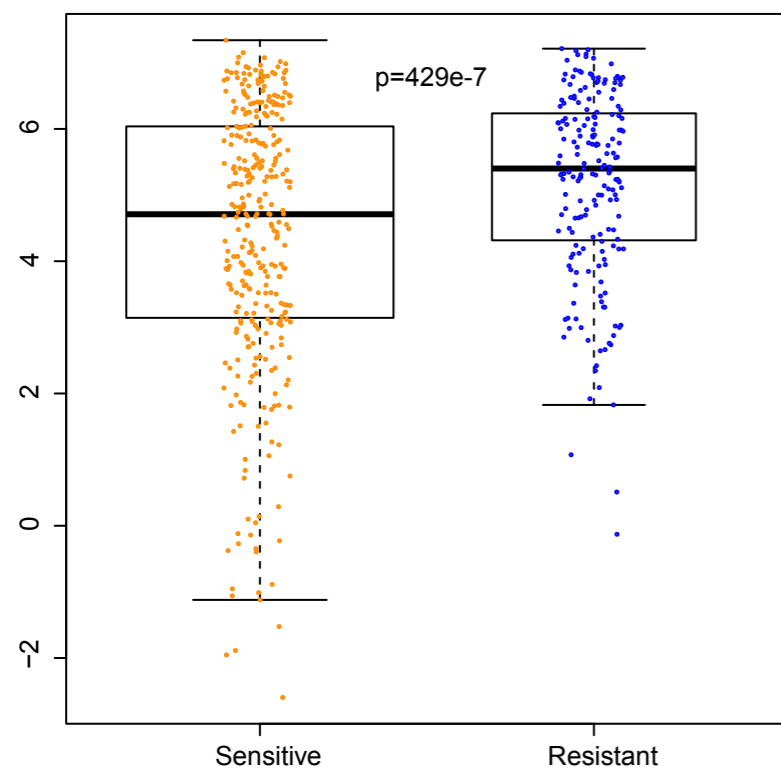

Paclitaxel

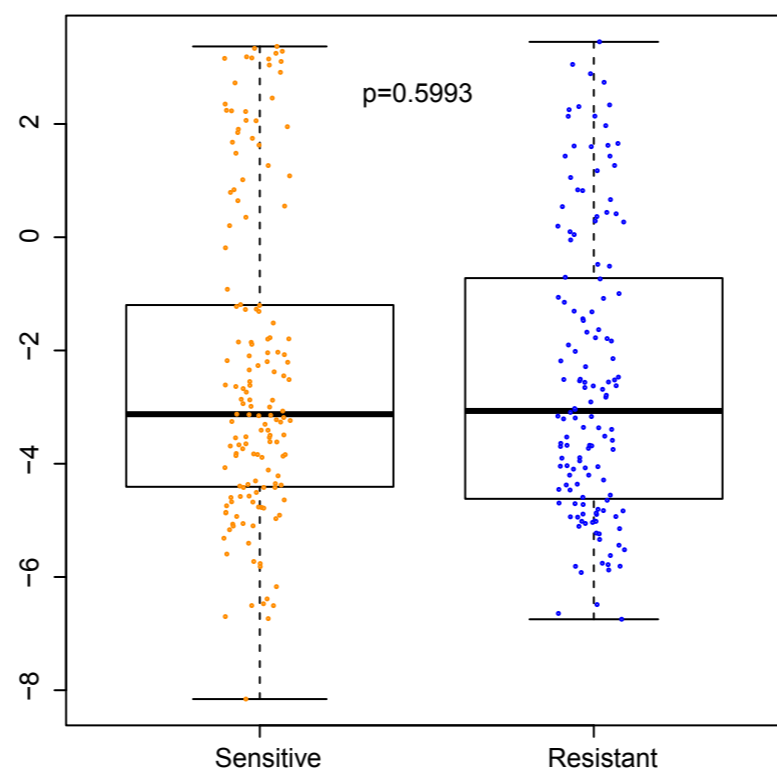

Sorafenib

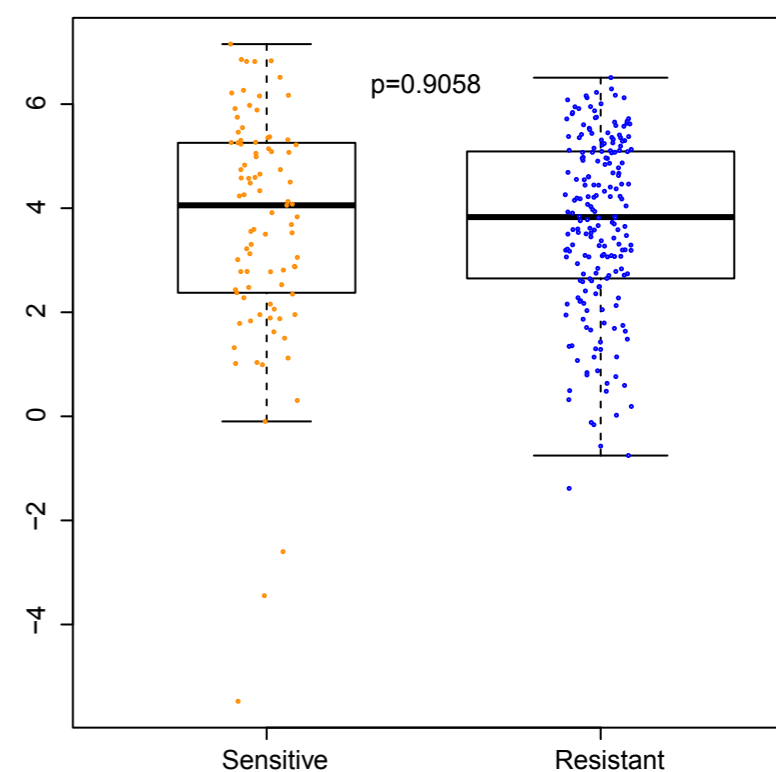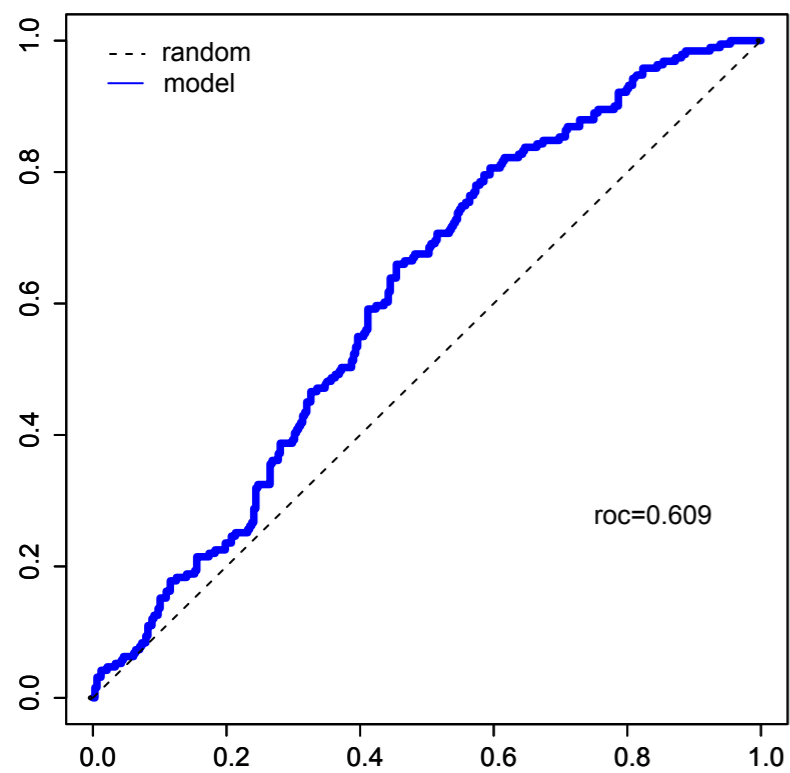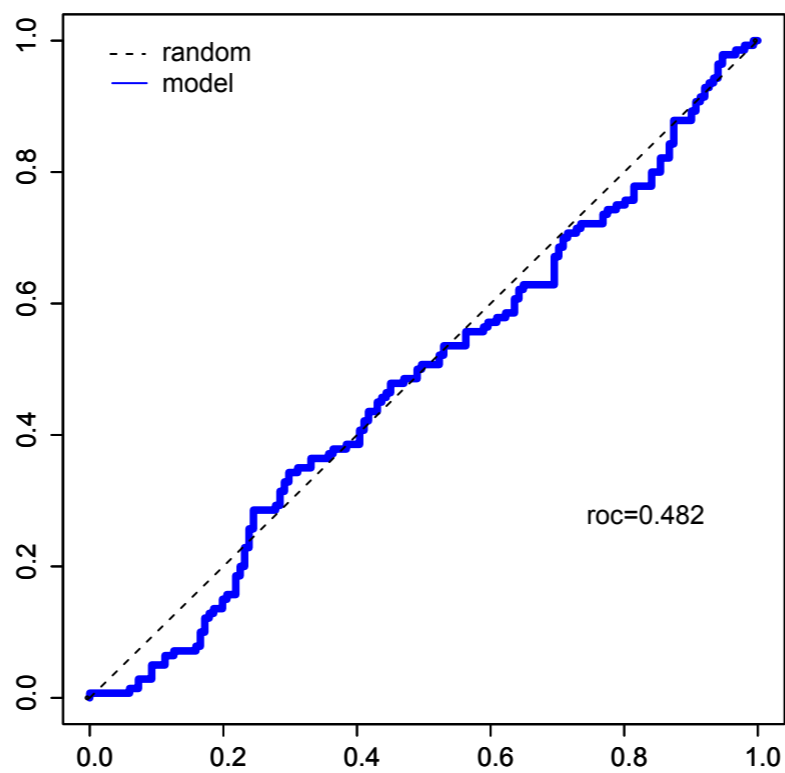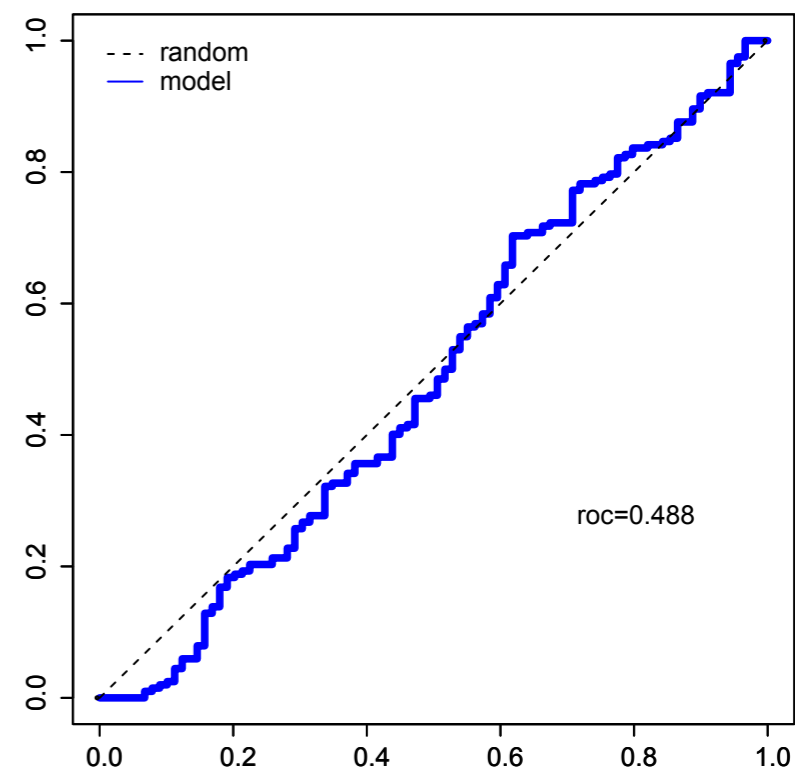

Supplement: Additional file 10: — Independent tests for PLX4720, Paclitaxel and Sorafenib in random forest predicting model. Boxplot and ROC curve (the bottom curve indicates drug response, measured as the area over the dose–response curve, i.e., activity area) have been built to evaluate the model. (A) For drug PLX4720, p-value by t test is 1.429e-7 and area under the curve is 0.609. (B) For drug Paclitaxel, p-value by t test is 0.5993 and area under the curve is 0.482. (C) For drug Sorafenib, p-value by t test is 0.9058 and area under the curve is 0.488. [file 12885_2015_1492_MOESM10_ESM.pdf]

PHA-665752

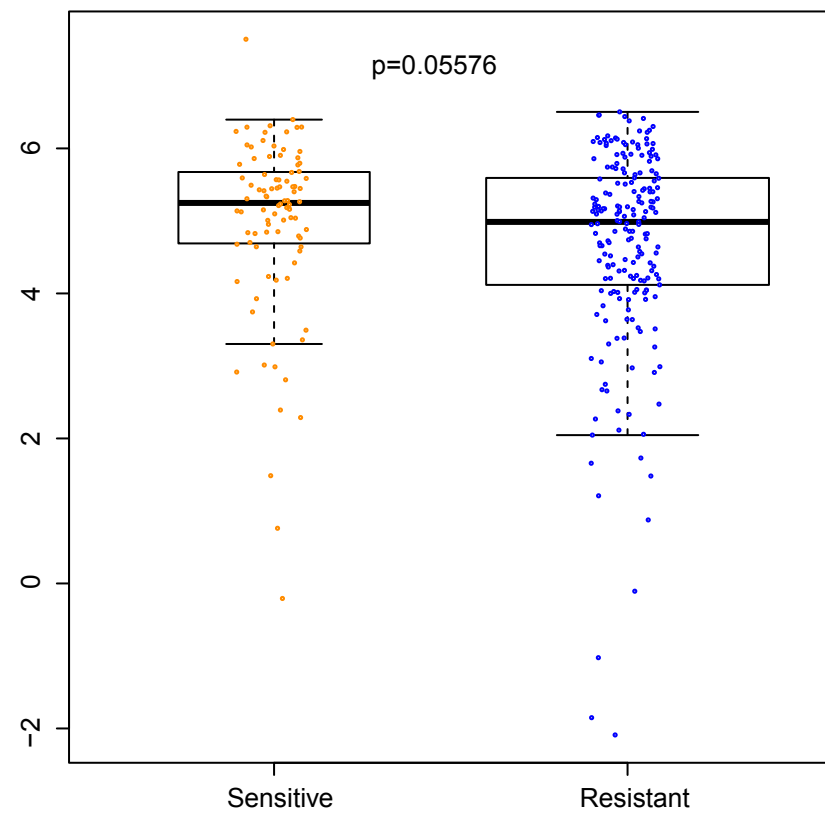

AZD0530

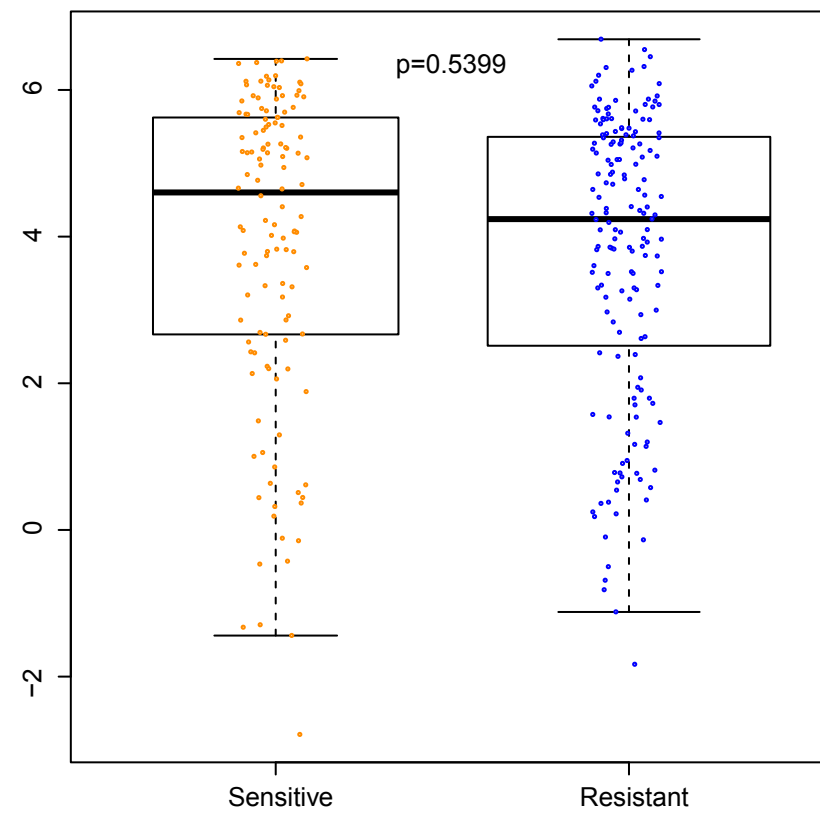

Paclitaxel

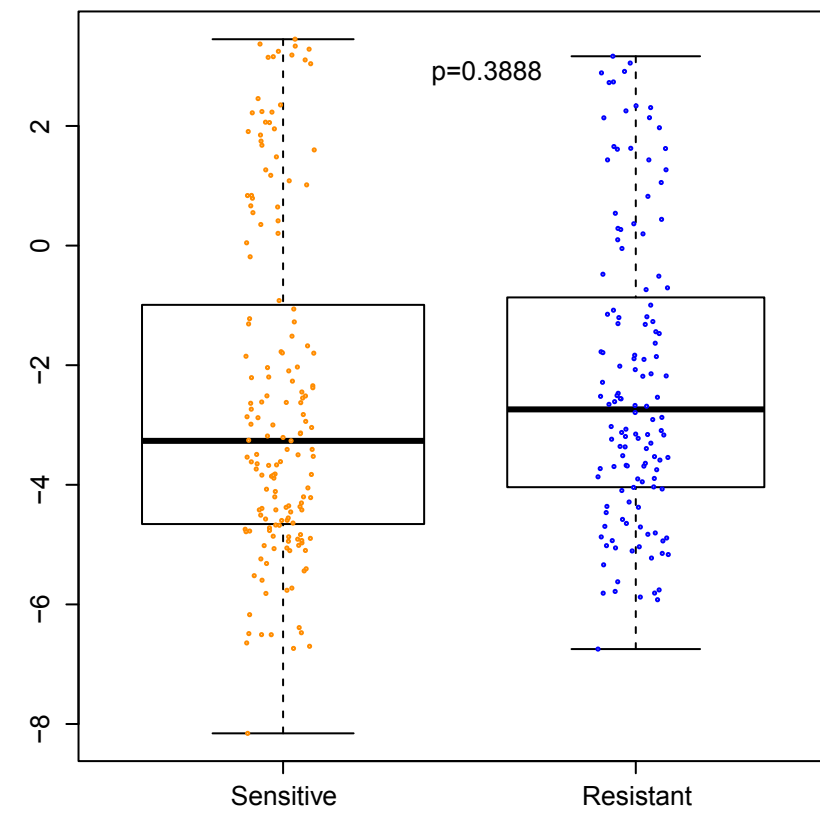

Sorafenib

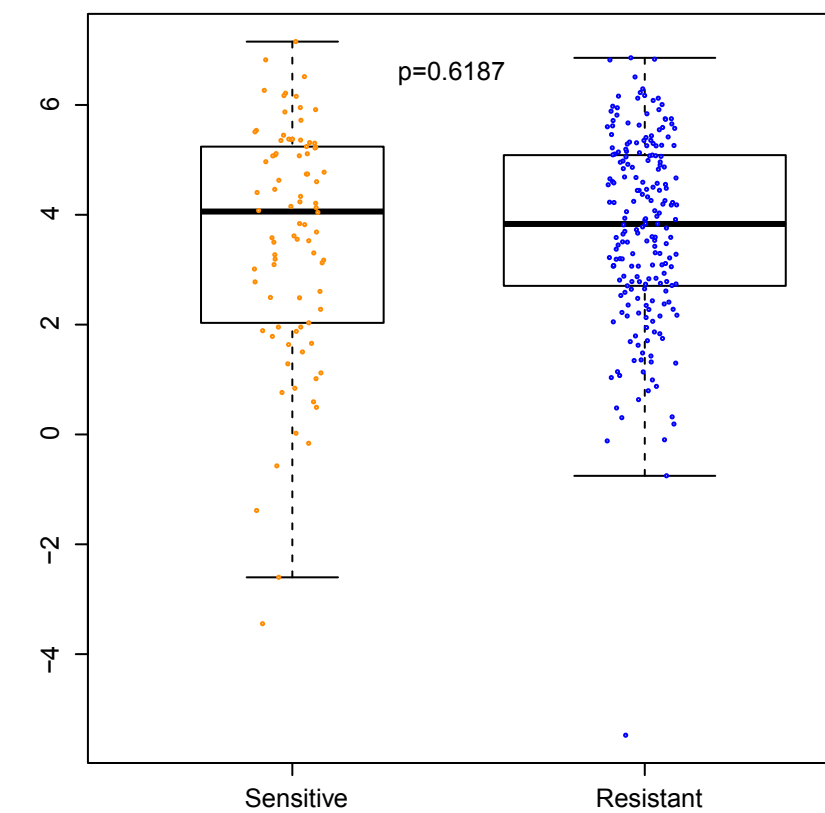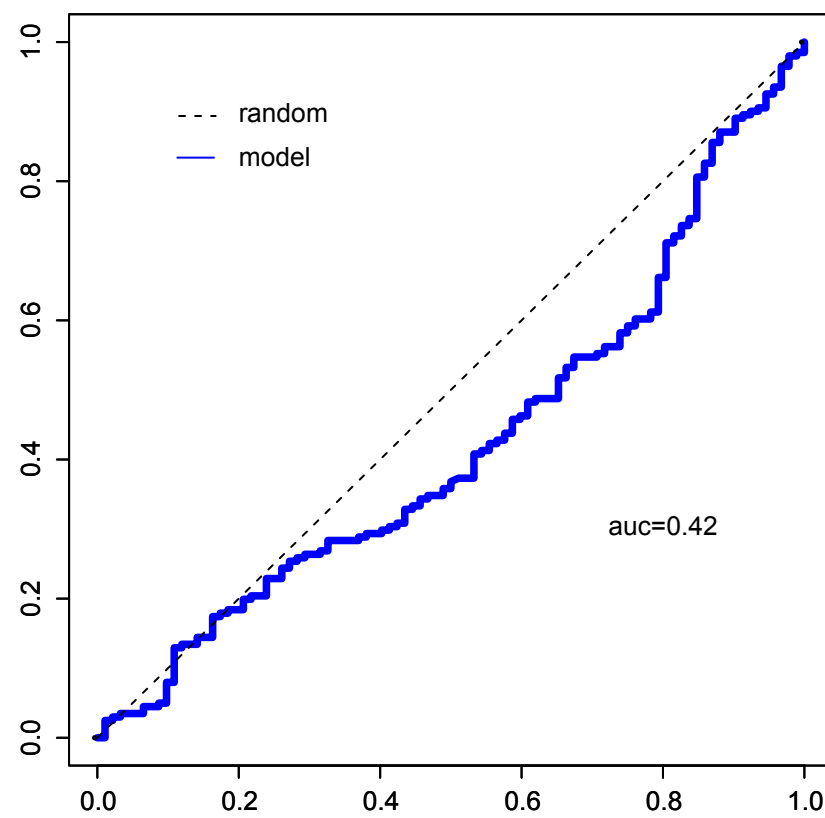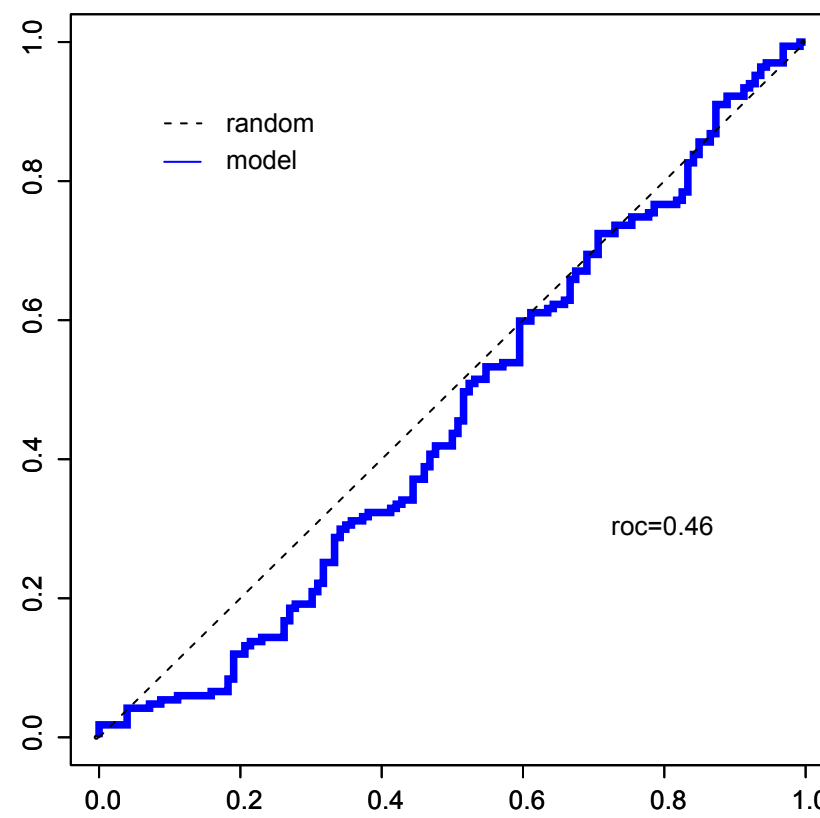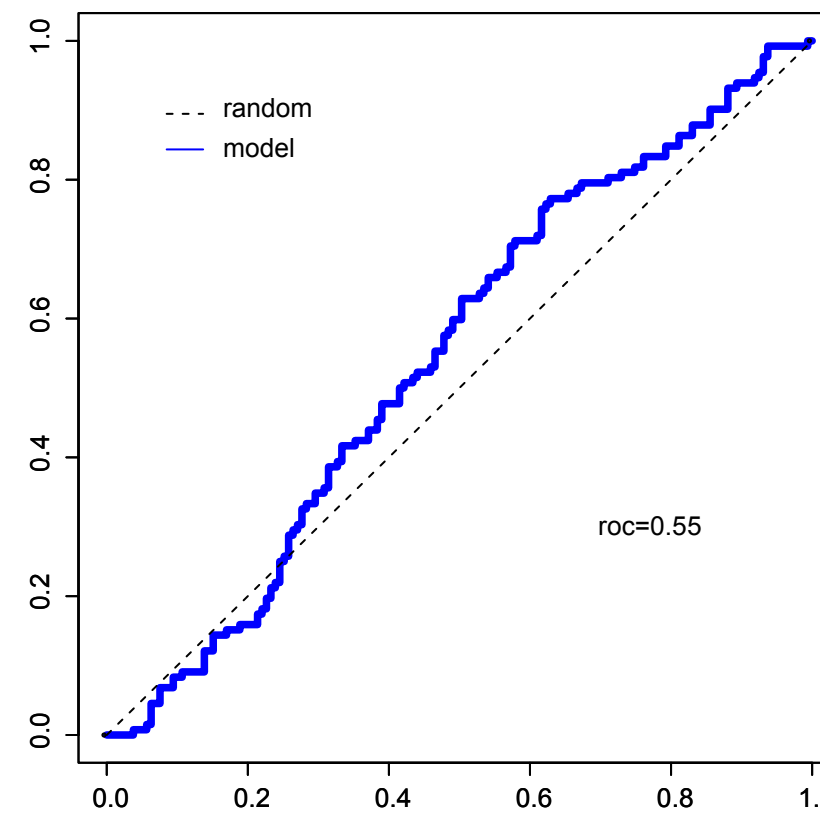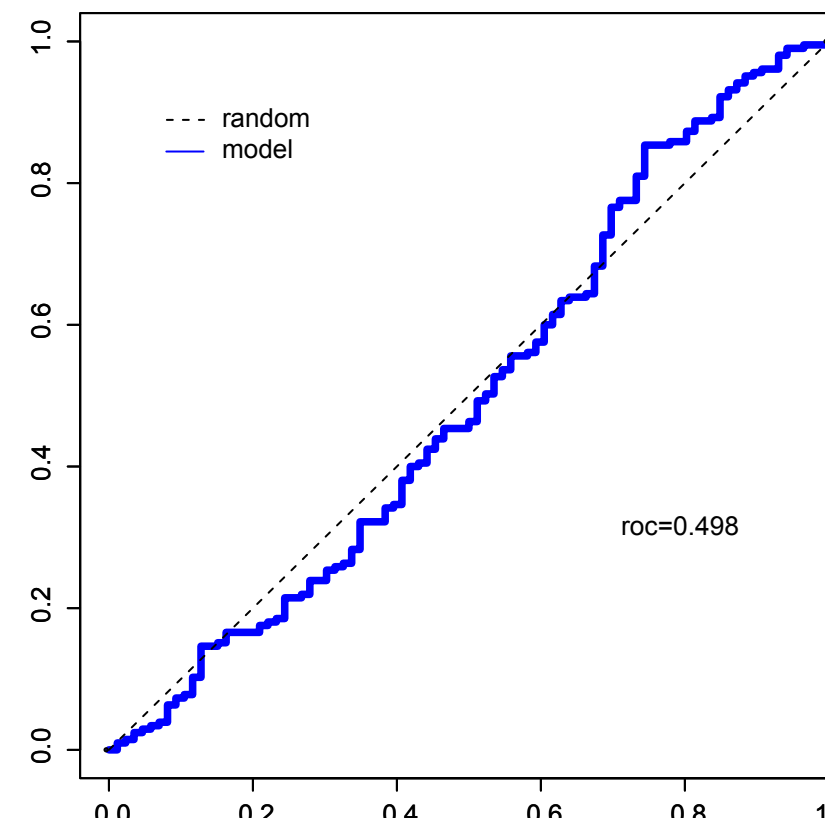

Supplement: Additional file 11: — Independent tests on CCLE model for PHA-665752, AZD0530, Paclitaxel and Sorafenib. The above graph (boxplot, roc curve) shows the performance of 4 drugs—PHA-665752, AZD0530, Paclitaxel and Sorafenib. [file 12885_2015_1492_MOESM11_ESM.pdf]

PD-0332991

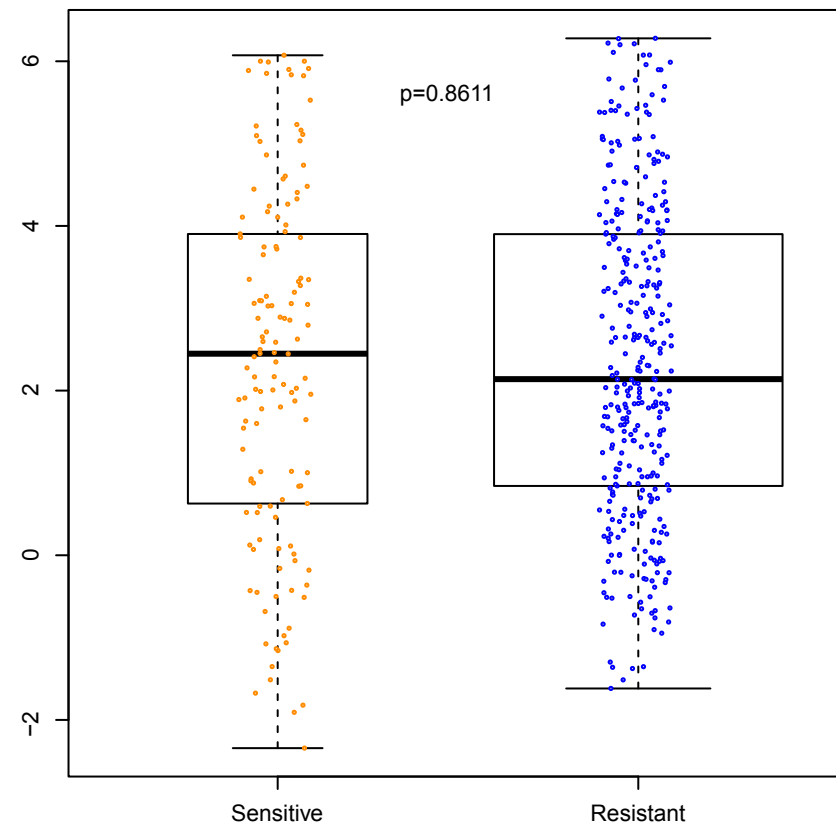

PLX4720

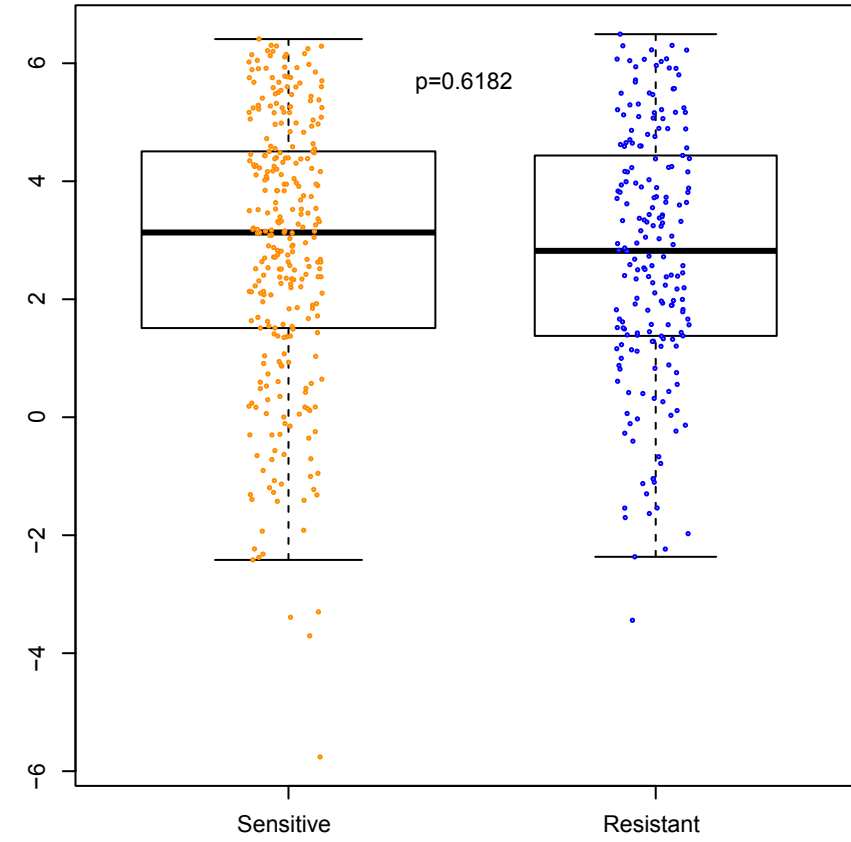

Lapatinib

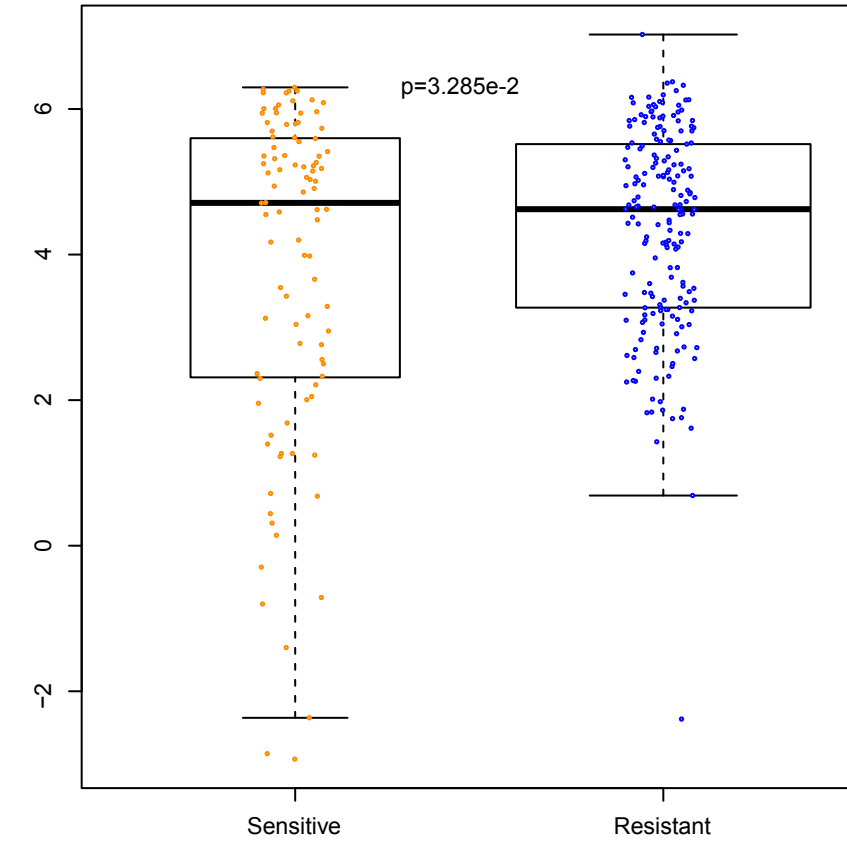

PF-2341066

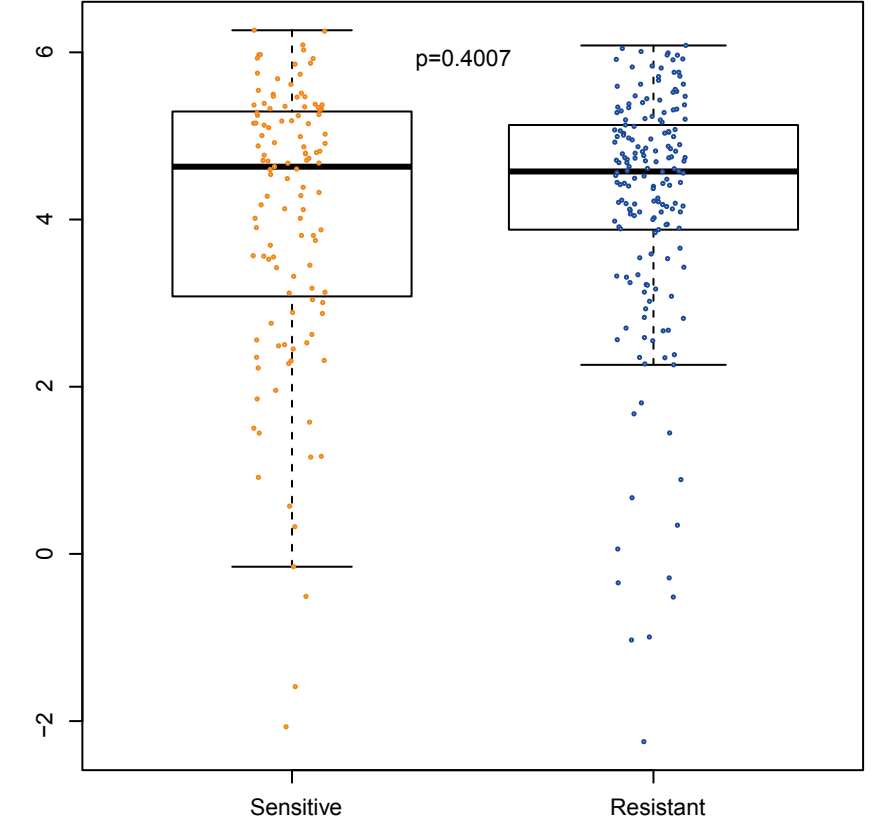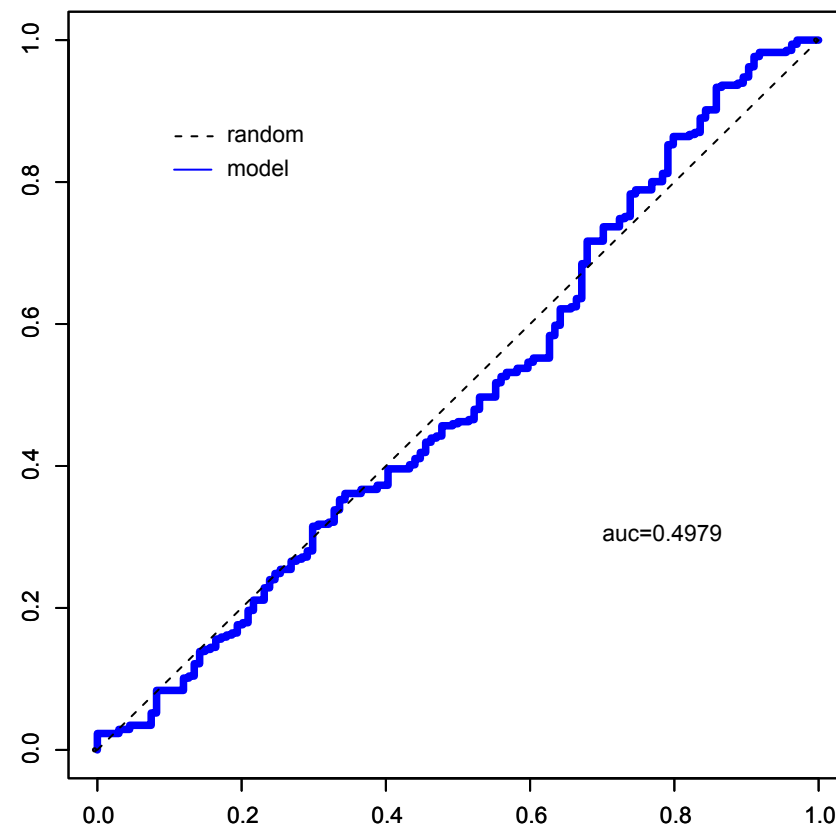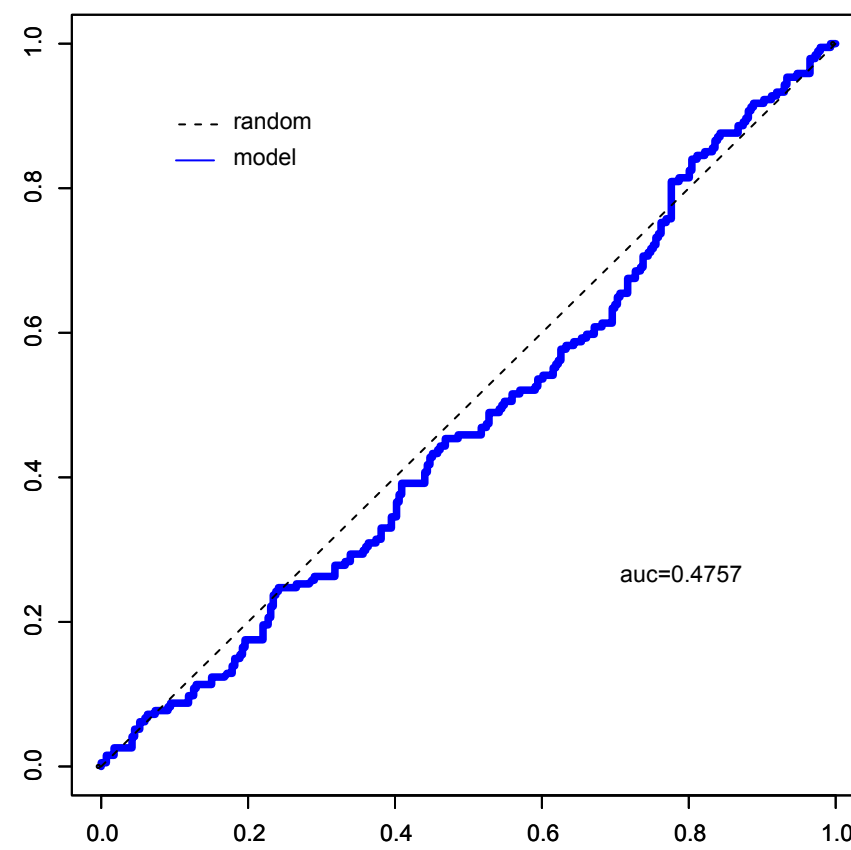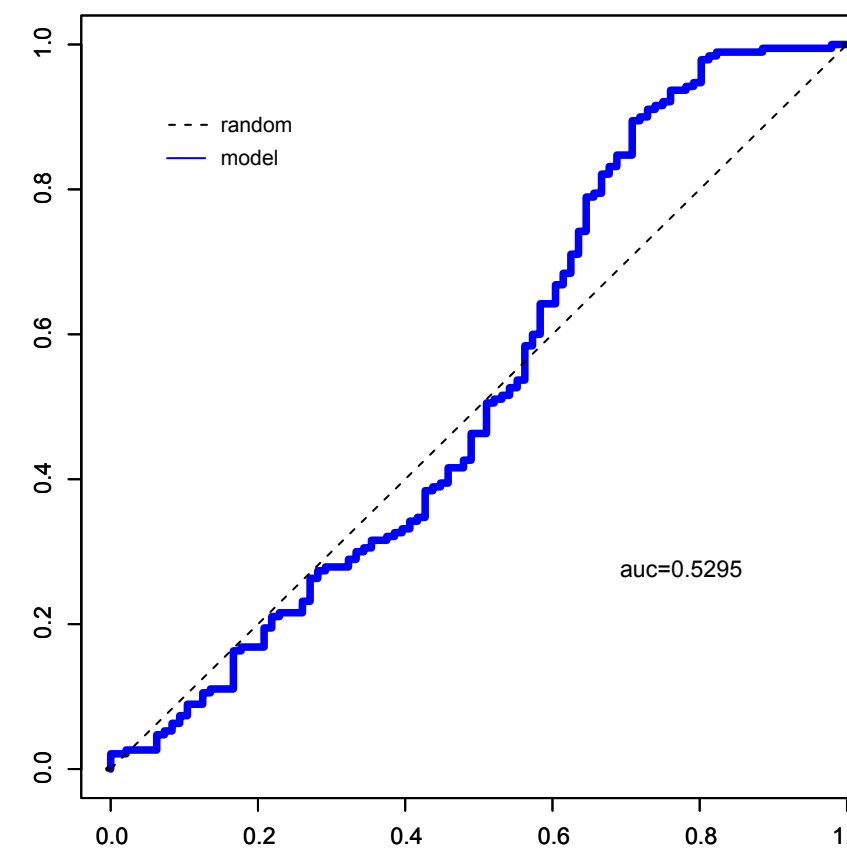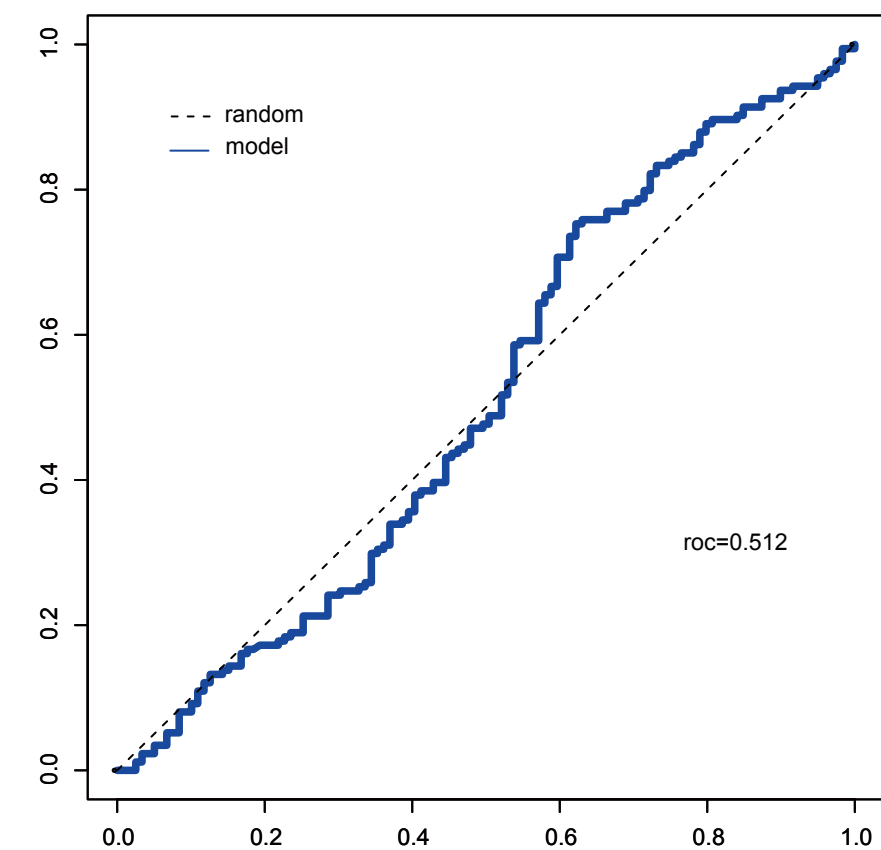

Supplement: Additional file 12: — Independent tests on CCLE model for PD-0332991, PLX4720, Lapatinib, and PF-2341066. The above graph (boxplot, roc curve) shows the performance of 4 drugs—PD-0332991, PLX4720, Lapatinib and PF-2341066. [file 12885_2015_1492_MOESM12_ESM.pdf]

AZD6244

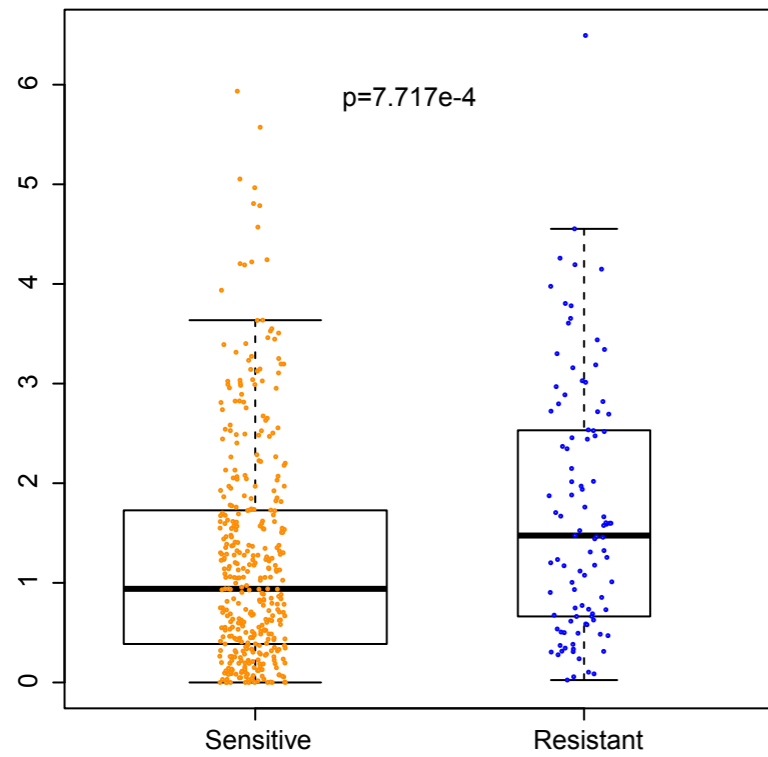

Erlotinib

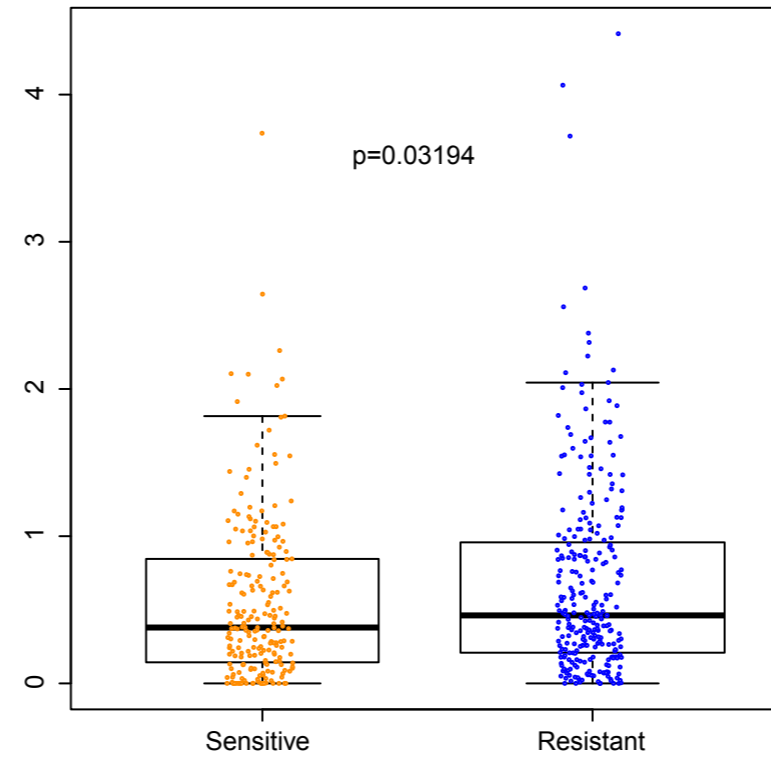

PD-0325901

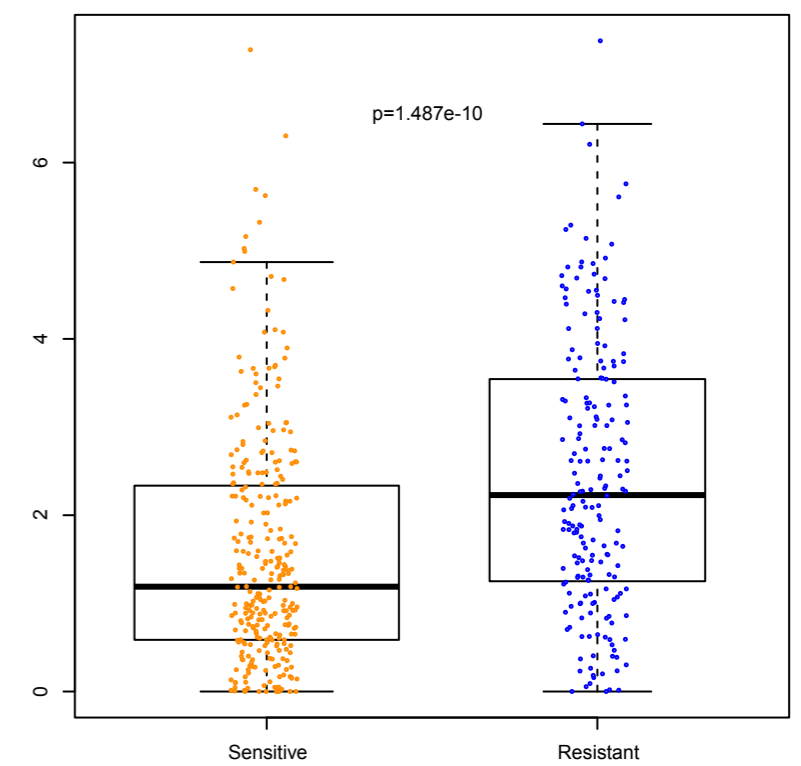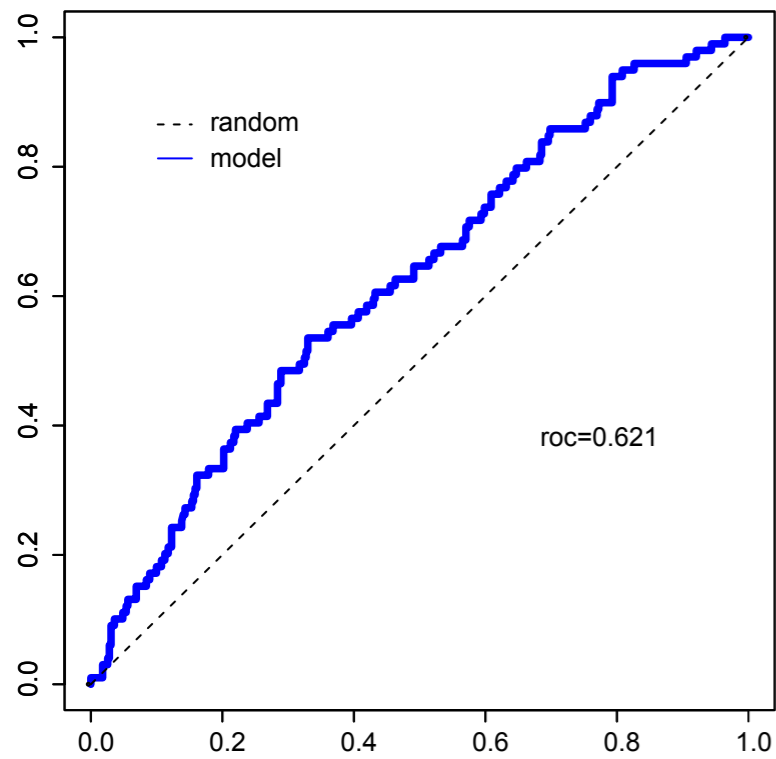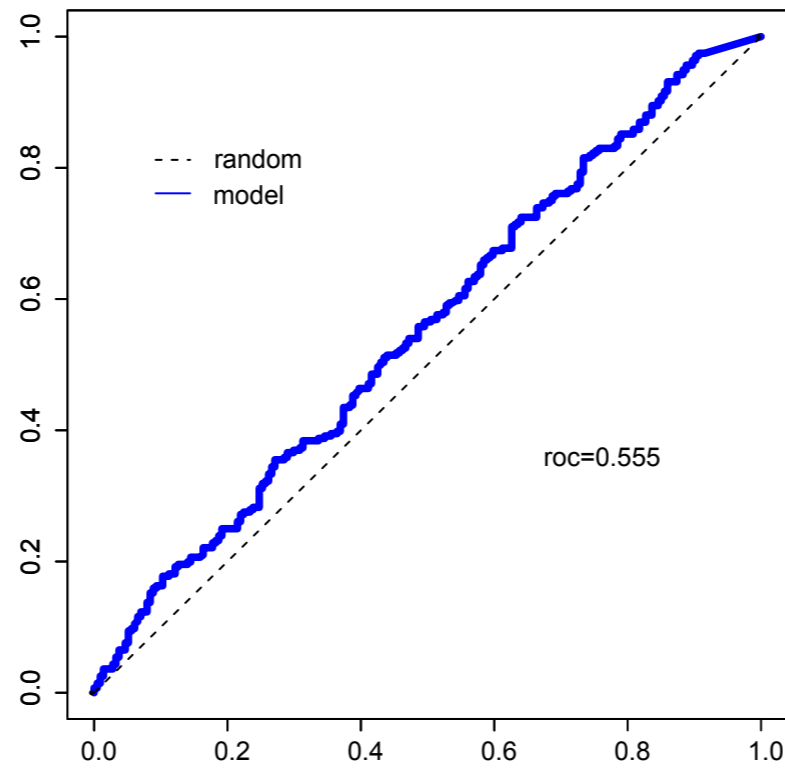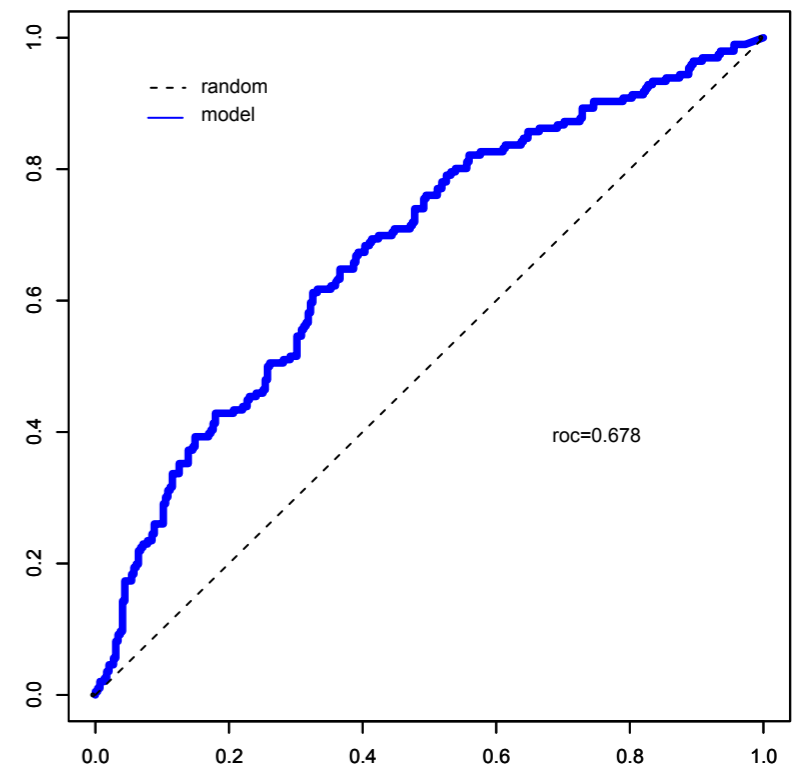

Supplement: Additional file 13: — Independent tests on CGP model for AZD6244, Erlotinib, PD-0325901. CGP IC50 data was used to build svm model, and then CCLE activity area data was used to test the model. Boxplot and ROC curve have been built to evaluate the svm model. For drug AZD6244, p-value by t test is 7.717e-4 and area under the curve is 0.621. For drug Erlotinib, p-value by t test is 0.03194 and area under the curve is 0.555. For drug PD-0325901, p-value by t test is 1.487e-10 and area under the curve is 0.678. [file 12885_2015_1492_MOESM13_ESM.pdf]

Results of cross-validation(EXP vs EXP+CPV+SNP)

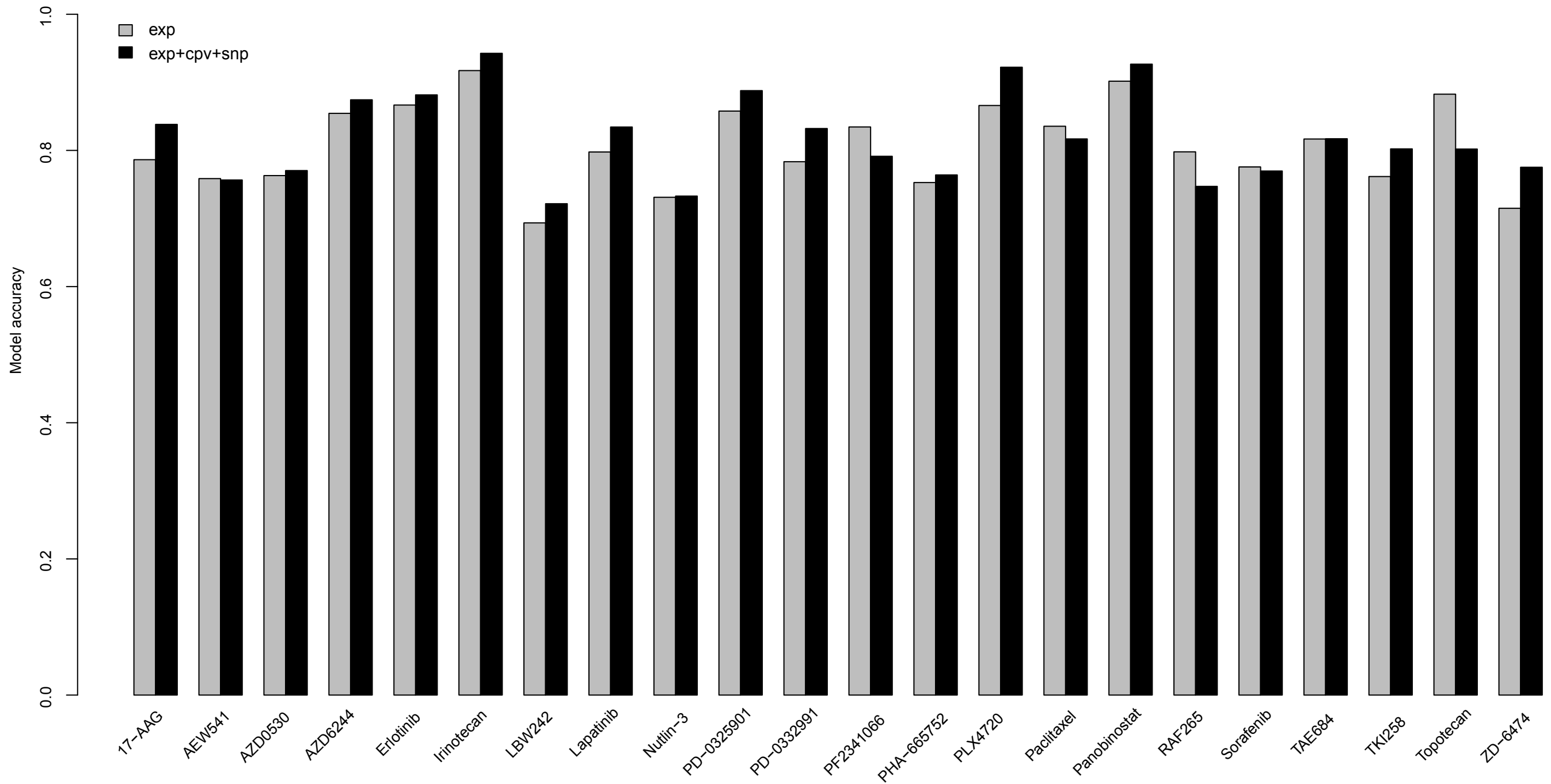

Supplement: Additional file 14: — Results of cross validation between different data sets (EXP vs EXP + CPV + SNP). Gene expression, copy number and gene mutation data sets were combined into an integrated data sets. Then this integrated data sets was used to conduct SVM-RFE and feature selection. Consequently selected features were used to build SVM model. For each drug in CCLE, 10-fold cross validation was performed to test the robustness of the model. Comparison of cross validation results between different data sets (EXP vs EXP + CPV + SNP) can be seen in this barplot. [file 12885_2015_1492_MOESM14_ESM.pdf]
