# Supplementary material for: Anticancer drug sensitivity prediction in cell lines from baseline gene expression through recursive feature selection
Source: BMC Cancer. 2015 Jun 30;15:489. doi: 10.1186/s12885-015-1492-6 (PMC4485860; doi:10.1186/s12885-015-1492-6)
Supplement: Additional file 5: — Relationships between selected features and cancer. For drug AZD6244, Erlotinib and PD-0325901, functions of selected genes in tumorigenesis are listed here. Many selected genes are reported to have close relationship with tumorigenesis or cancer progression. (A) Relationships between selected features and cancer for drug AZD6244. (B) Relationships between selected features and cancer for drug Erlotinib. (C) Relationships between selected features and cancer for drug PD-0325901. [file 12885_2015_1492_MOESM5_ESM.docx]

**(A)**

**AZD6244**

| ***Impor genes*** | ***Description*** |
| --- | --- |
| ***SPRY2*** | An inhibitor of mitogen-activated protein kinase signaling, is down-regulated in hepatocellular carcinoma. In the research performed by Chee Wai Fong et al, they observed a consistent reduced expression of the Spry2 transcript and protein in malignant hepatocytes compared with normal or cirrhotic hepatocytes in human hepatocellular carcinoma where the MAPK (mitogen-activated protein kinase) activity is enhanced via multiple hepatocarcinogenic factors. |
| ***GDF15*** | In the research performed by Vera L. Costa et al, which aims to identify a panel of epigenetic biomarkers for accurate bladder cancer detection in urine sediments, they found that three Epigenetic Biomarkers (GDF15, TMEFF2, and VIM) can predict bladder cancer from DNA-Based analyses of Urine samples accurately. |
| ***ITGA4*** | In the study performed by ZHEN-QIANG LIAN et al, they detected 70 significantly hypermethylated genes in breast cancer tissue, including this novel hypermethylated gene (ITGA4). Direct bisulfite sequencing showed widespread methylation occurring in intragenic regions of the WT1, PAX6and ITGA4 genes and in the promoter region of the OTX2 gene in breast cancer tissues. |
| ***DAB2*** | Human Doc-2/DAB2 interactive protein gene (hDAB2IP), located on chromosome 9q33.1-q33.3, is a novel member of the Ras GTPase-activating family. It interacts directly with disabled 2 protein (DAB2; also known as DOC-2, differentially expressed in ovarian carcinoma which appears to be a tumor suppressor in malignant cells, including mammary, prostate, and ovarian cancers. HDAB2IP and DOC-2/DAB2 form a unique protein complex and have a negative regulatory activity to the Ras-mediated signal pathway. |
| ***PPAP2C*** | The protein encoded by this gene is a member of the phosphatidic acid phosphatase (PAP) family. PAPs convert phosphatidic acid to diacylglycerol, and function in de novo synthesis of glycerolipids as well as in receptor-activated signal transduction mediated by phospholipase D. This protein is similar to phosphatidic acid phosphatase type 2A (PPAP2A) and type 2B (PPAP2B). All three proteins contain 6 transmembrane regions, and a consensus N-glycosylation site. This protein has been shown to possess membrane associated PAP activity. Three alternatively spliced transcript variants encoding distinct isoforms have been reported. |
| ***AXL*** | Axl, also called UFO, ARK, and Tyro7, was originally identified as a transforming gene in human leukemia. The 894-amino-acid human protein has a mass of 140 kDa, with roughly equal distribution of amino acids on either side of the plasma membrane. The ligand of Axl, Gas6 protein, is so named by virtue of the initial finding that the gene (growth arrest-specific gene 6) that encodes the protein is highly expressed in growth-arrested cells. The biologic function of Axl/Gas6 in normal and malignant cell biology has not been completely elucidated, but appears to be complex. |

**(A)** Relationships between selected features and cancer for drug AZD6244.

**(B)**

**Erlotinib**

| ***Impor Genes*** | ***Description*** |
| --- | --- |
| ***CYP1A1*** | In patients with breast cancer there is a correlation between the CYP1A1 CC allele and some factors indicating poor prognosis, including more lymph node metastases as well as a more advanced clinical stage. |
| ***RASSF2*** | 1. In a research performed by Kimishige Akino et al, they found that the Ras Effector RASSF2 is a novel tumor suppressor gene in human colorectal cancer.  2. RASSF2, a member of the RAS association domain family 1 (RASSF1), is a candidate tumor suppressor gene (TSG) that is silenced by promoter hypermethylation in several human cancers. In a research performed by KYOICHI KAIRA et al, they examined the expression of RASSF2 mRNA and the promoter methylation status in lung cancer cell lines and in tumor samples of 106 primary non-small cell lung cancers by methylation-specific PCR. Their results indicate that aberrant methylation of the RASSF2 gene with the subsequent loss of RASSF2 expression plays an important role in the pathogenesis of lung cancers. |
| ***KLF11*** | Epigenetic inactivation and subsequent transcriptional repression of the KLF11 gene is quite frequent in myelodysplastic syndromes. Patients with an isolated 5q-deletion seem to harbour a distinct epigenetic profile. |

**(B)** Relationships between selected features and cancer for drug Erlotinib.

**(C)**

**PD-0325901**

| **Impor Genes** | **Description** |
| --- | --- |
| SLC34A2 | In a research conducted by Soares IC *et al*, they found higher expression of NaPi2b (SCL34A2) in ovarian carcinoma than in normal tissue. Moreover, a comprehensive analysis indicates that SCL34A2 is the only gene of the several phosphate transporters genes whose expression differentiates normal from carcinoma samples, suggesting it might exert a major role in ovarian carcinomas. (Soares IC *et al, 2012*) |
| DUSP6 | Dual‑specificity phosphatase 6 (DUSP6), a specific negative feedback regulator of phosphorylated extracellular signal‑regulated kinase, was found to play an important role in numerous types of solid tumors as a tumor suppressor. In a study focused on DUSP6 performed by JIANJUAN MA *et al* , their results support the role of DUSP6 as a novel candidate tumor suppressor gene in ESCC (esophageal squamous cell carcinoma), which may be a potential prognostic marker for ESCC. (JIANJUAN MA *et al,* 2013) |
| LYN | Lyn is a target gene for prostate cancer: sequence-based inhibition induces regression of human tumor xenografts.(Mirela Goldenberg-Furmanov *et al,* 2004) |
| HMMR | A recent study conducted by Bolot Kalmyrzaev *et al* used a network modeling strategy to generate a set of genes linked by potential functional associations. The hyaluronan-mediated motility receptor (HMMR) gene was identified as being as functionally associated with BRCA1 and thus a candidate breast cancer gene. (Bolot Kalmyrzaev, Paul D.P. Pharoah, Douglas F. Easton, et al, 2008) |
| LGR5 | In a research conducted by Yi-shiuan Liu et al, they found that forced expression of Lgr5 increased the CRC sphere-forming efficiency and spheroid size while depletion of Lgr5 reduced the stem cell property in cultured CRC cells. Over-expression of Lgr5 also reduced the sensitivity of cultured CRC cells, including adherent and spheroids, towards 5-fluoracil and oxalipatin. In addition, Lgr5 positively regulates the expression of ABCB1 in both adherent and spheroid CRC cells. Finally, in human CRC tissues, higher expression levels of Lgr5 were associated with higher ABCB1 expression. (Yi-shiuan Liu et al, 2013) |

**(C)** Relationships between selected features and cancer for drug PD-0325901.
